# Beyond the boundaries of microbial diversity in radioactive environments: novel microbial species isolated from a former silver-uranium mine’s radon-saturated waters

**DOI:** 10.1186/s12866-026-05379-9

**Published:** 2026-07-13

**Authors:** Gabriela Kapinusova, Kunal Jani, Petr Pajer, Alena Filipova, Pavel Ulbrich, Tomas Cajthaml, Michal Strejcek, Jachym Suman, Ondrej Uhlik

**Affiliations:** 1 Department of Biochemistry and Microbiology, Faculty of Food and Biochemical Technology, University of Chemistry and Technology, Prague, Prague, Czech Republic; 2 Institute of Evolutionary Ecology and Conservation Genomics, Universität Ulm, Ulm, Germany; 3 Military Health Institute, Ministry of Defence of the Czech Republic, Prague, Czech Republic; 4 Institute of Microbiology of the Czech Academy of Sciences, Prague, Czech Republic

**Keywords:** Microbial diversity, Novel bacterial taxa, Phylogenetic novelty, Mineral springs, Radon-saturated waters

## Abstract

The Svornost mine in Jáchymov (Joachimstahl), Czech Republic, historically the world’s first radium mine and formerly a silver-uranium mine, is currently the deepest facility exploiting radon-saturated waters. These geologically ancient waters, approximately 13,000 years old, exhibit unique physicochemical properties and are generally considered inhospitable to life. Nevertheless, they harbor phylogenetically and evolutionarily distinct microorganisms. A previous culturomic survey yielded nine Gram-stain-negative, rod-shaped, aerobic bacterial isolates. In this study, a polyphasic taxonomic approach, including 16S rRNA gene phylogeny, core-genome analyses, and comparisons of genome indices such as average nucleotide identity (ANI), average amino acid identity (AAI), and percentage of conserved proteins (POCP), together with physiological and chemotaxonomic characterization, confirmed the taxonomic novelty at species level of these nine strains. The novel strains span five bacterial classes (α-, β-, and γ-*Proteobacteria*, *Cytophagia*, and *Flavobacteriia*) and eight genera, and are herein proposed as *Hymenobacter abyssi* J193^T^ (= DSM 113871^T^ =CCM 9301^T^), *Rhizobacter abyssi* J219^T^ (= DSM 113781^T^ =CCM 9293^T^), *Aquabacterium abyssi* J223^T^ (= DSM 113847^T^ =CCM 9303^T^), *Aquincola abyssi* J276^T^ (= DSM 113828^T^ =CCM 9294^T^), *Flavobacterium abyssi* J372^T^ (= DSM 113872^T^ =CCM 9299^T^), *Phenylobacterium abyssi* J367^T^ (= DSM 113838^T^ =CCM 9302^T^), *Phenylobacterium fontinale* J426^T^ (= DSM 113839^T^ =CCM 9296^T^), *Mesorhizobium abyssi* J428^T^ (= DSM 114114^T^ =CCM 9297^T^) and *Pseudomonas fontinalis* J452^T^ (= DSM 113783^T^ =CCM 9298^T^). These findings highlight the potential of radon-saturated ancient waters as a source of novel microbial diversity, offering insights into the evolution of bacteria under extreme, spatially isolated environmental conditions.

**Supplementary Information:**

The online version contains supplementary material available at https://doi.org/10.1186/s12866-026-05379-9.

## Background

Remote and pristine environments have recently gained increased attention as valuable sources of phylogenetically novel lineages and microorganisms possessing unique metabolic and ecological traits [1–4]. Such ecosystems, often characterized by extreme physical or chemical conditions, provide selective pressure that provokes microbial adaptation and diversification. However, the microbial diversity of such habitats remains largely unknown and many of the phylogenetic branches found in these habitats are represented by few or no cultured isolates.

One such intriguing site is the former silver and uranium mine in Jáchymov, Czech Republic, where four springs with elevated concentrations of radon and other dissolved elements create specific geochemical conditions favoring the growth of previously undescribed microbial taxa. From a previous culturomic survey targeting microbial diversity in Jáchymov radon springs, nine phylogenetically novel bacterial isolates were successfully obtained [5]. The isolates represent five bacterial classes α-, β-, and γ-*Proteobacteria*, *Cytophagia*, and *Flavobacteriia* and belong to eight genera: *Hymenobacter*, *Mesorhizobium*,* Pseudomonas*, *Rhizobacter*, *Aquincola*,* Aquabacterium*,* Phenylobacterium* and *Flavobacterium*.

Members of the genus *Hymenobacter*, fam. *Hymenobacteraceae*, are typically red or pink pigmented bacteria that are often resistant to desiccation and UV radiation [6, 7]. They are widely distributed in terrestrial and extreme environments, including cold deserts, permafrost [7–9], and irradiated habitats [6, 10]. *Mesorhizobium*, fam. *Phyllobacteriaceae*, includes bacteria that are commonly capable of nitrogen fixation and are mostly associated with plant roots or rhizosphere environments. They can also be found in bare soil and aquatic ecosystems [11–13].

The genera *Pseudomonas* (fam. *Pseudomonadaceae*), and *Rhizobacter* with *Aquincola* (fam. *Burkholderiaceae*) comprise metabolically versatile, motile, aerobic bacteria capable of degrading a variety of aromatic compounds and xenobiotics. They inhabit soil, water, rhizosphere, and host-associated niches where they play important roles in bioremediation, plant growth promotion and nitrogen fixation [14–19]. Filamentous bacteria of *Aquabacterium* [20–22], fam. *Sphaerotilaceae*; stalked/prosthecate cells of *Phenylobacterium* [23–25], fam. *Caulobacteraceae*; and yellow-pigmented members of *Flavobacterium* [26–28], fam. *Flavobacteriaceae*, are usually abundant in aquatic and soil habitats where they contribute to the degradation of complex organic polymers and biofilm formation.

In the present study, these nine novel Gram-stain negative isolates are subjected to comprehensive phenotypic, chemotaxonomic, and genomic characterization in order to clarify their taxonomical categorization, and ultimately, nine novel species are proposed, with these isolates as their type strains.

## Methods

### Site description and bacterial isolation

All the strains described in this study were isolated during a comprehensive microbial analysis of radon-saturated springs at the former silver-uranium Svornost mine in Jáchymov, Czech Republic (50°22′22.9″N 12°54′42.1″E). Strain J193^T^ was isolated from the spring Agricola (water yield 10 l/min., temperature 29 °C, radioactivity 20 kBq/l); strains J219^T^, J223^T^ and J276^T^ from the spring Běhounek (300 l/min., 36 °C, 10 kBq/l); strains J367^T^, J372^T^ were isolated from the spring C1 (30 l/min., 29 °C, 11 kBq/l) and strains J426^T^, J428^T^, J452^T^ from the spring Curie (30 l/min., 29 °C, 5 kBq/l) by using an inoculum created by concentrating 20 l of every spring water into 25 ml. The detailed isolation process and subsequent strain acquisition is described in Kapinusova et al. 2022 [5].

### Taxonomic classification of novel strains

#### 16S rRNA gene phylogeny

Preliminary taxonomic assignments of novel strains were inferred from 16S rRNA gene phylogenetic analysis [29]. For genomic DNA extraction, strains were cultivated for 24–96 h on solid media according to strain preference (J193^T^, J367^T^, J372^T^, J426^T^, J452^T^ on 1/10 PCA, 24–96 h, 28 °C; strains J219^T^, J223^T^, J276^T^ on R2A, 24–48 h, 37 °C; strain J428^T^ on R2A, 72 h, 28 °C). The isolated pure cultures were subsequently harvested, and the genomic DNA was extracted using the PureLink™ Genomic DNA Kit (Invitrogen) in accordance with the manufacturer’s instructions.

Amplification of the 16S rRNA gene was performed using the universal primers 8–27F (5´-AGAGTTTGATCMTGGCTCAG-3´) [30] and 1509–1492R (5´-GYTACCTTGTTACGACTT-3´) [30]. The preparation of the reaction mix, PCR conditions and the sequencing procedure of the resulting amplicons are described in Kapinusova et al., 2022 [5]. All sequences were quality-checked, trimmed, and aligned using MEGA X [31]. Nearly complete 16S rRNA gene sequences (> 1300 bp; Table 1) were compared against the EzBioCloud 16S rRNA gene sequence database [32] to determine the closest phylogenetic relatives and assess sequence similarity.

**Table 1:** The closest type strains based on the 16S rRNA gene sequence similarity

Strain	Sequence length (bp)	Closest type strain based on the 16S rRNA gene sequence similarity	Hit (%)	Closest type strain with available genome based on the 16S rRNA gene sequence similarity	Hit (%)
J193	1344	*Hymenobacter guriensis* BT594^T^	99.78	*Hymenobacter lutimineralis* KIGAM108 ^T^	98.07
J219	1402	*Schlegelella koreensis* ID0723^T^	98.08	*Piscinibacter caeni* MQ-18^T^	97.71
J223	1394	*Aquabacterium humicola* RJY3^T^	98.06	*Aquabacterium terrae* S2^T^	97.85
J276	1385	*Aquincola agrisoli* MAHUQ-54^T^	98.92	*Aquincola agrisoli* MAHUQ-54^T^	98.92
J367	1363	*Phenylobacterium immobile* strain E^T^	98.24	*Phenylobacterium immobile* strain E^T^	98.24
J372	1366	*Flavobacterium album* HYN0059^T^	97.37	*Flavobacterium album* HYN0059^T^	97.37
J426	1359	*Phenylobacterium muchangponense* A8^T^	98.31	*Phenylobacterium zucineum* HLK1^T^	97.35
J428	1339	*Mesorhizobium qingshengii* CCBAU 33,460^T^	98.30	*Mesorhizobium qingshengii* CCBAU 33,460^T^	98.30
J452	1409	*Pseudomonas campi* S1-A32-2^T^	99.86	*Pseudomonas campi* S1-A32-2^T^	99.86

To elucidate the evolutionary relationships between the novel taxa and their closest type strains, more than 20 of the most similar 16S rRNA gene sequences were retrieved from the EzBioCloud database and aligned based on their secondary structures using EzEditor2 [33]. Phylogenetic trees were subsequently reconstructed in MEGA 11 [34] employing the Kimura two-parameter model [35] and the secondary structure-aligned sequences. Both Neighbor-Joining [36] and Maximum-Likelihood [37] algorithms were used to infer phylogenetic relationships. The robustness of the tree topologies was evaluated by bootstrap analysis with 1,000 replications, and the percentage of replicate trees in which the associated taxa clustered together was indicated. Evolutionary rate heterogeneity among sites was modeled using a discrete gamma distribution with five categories.

#### Genomic analysis

Genomic analysis was conducted as described by Kapinusova et al. [5]. Briefly, the high-quality DNA was processed for the whole genome library preparation according to Lopez-Echartea et al., 2021 [38]. The obtained libraries were pooled in equimolar concentrations prior to sequencing on an Oxford Nanopore Technologies MinION platform using a FLO-MIN106 flow cell. Guppy (v3.2.10) was used to perform the base-calling of nanopore sequencing signals and Porechop- and Filtlong-based for quality filtering. The quality-filtered reads (≤ 500 bp) were used for genome assembly using the Canu assembler (v1.7) [39]. The polishing of the Canu assemblies was carried out using Medaka (v1.4.3) (https://github.com/nanoporetech/medaka). CheckM, available on the Kbase server, was used to assess the quality and the completeness of the genome assemblies [40, 41]. Genome annotations for the inference of the functional potential of the bacteria were carried out using the combination of Prokka and PATRIC online servers [42–44]. The genome assembly and annotation processes are described in more detail elsewhere [5].

The phylogenetic relationships between the studied strains and their closest relatives were further inferred using the UBCG pipeline, which employs a core gene-based phylogenomic approach [45]. The UBCG analyses were based on a concatenated alignment of 92 conserved single-copy housekeeping genes [5]. To further assess the taxonomic novelty of the isolates, genome-wide similarity indices were calculated, including average nucleotide identity (OrthoANI), average amino acid identity (AAI), and the percentage of conserved proteins (POCP). These metrics were obtained using the EzGenome ANI calculator [46], EzAAI [47], and Galaxy Protologger [48].

### Physiology and chemotaxonomy

#### Microscopic and macroscopic morphology

Macroscopic characteristics (color, shape and surface) of all strains were assessed on solid media (R2A or 1/10 PCA or 1/5 TSA according to each strain’s preference). Transmission electron microscopy was used to examine microscopic characteristics such as cell shape, cell size, presence of flagella and other extracellular structures. Standard Gram-staining procedures as well as the KOH string test were used for Gram-stain examination.

#### Determination of growth requirements

The temperature dependence of growth was studied on solid media inoculated and placed in different incubators (4, 8, 12, 16, 20, 24, 28, 32, 37, 42, 45 °C) to determine the temperature range of each strain’s growth ability; and in liquid media cultured in a narrower temperature range (26, 28, 32, 34, 37 °C) for 24–72 h to determine the optimum growth temperature. Growth capability in the presence of NaCl (0.1, 0.5, 1, 1.5, 2, 2.5, 3, 3.5, 4, 5, 6, 7%_w/v_) was determined using a series of solid media with adjusted NaCl concentrations. Similarly, solid media with adjusted pH (using 0.5 M phosphate, citrate, or glycine buffer) were used to determine the pH optimum for growth (4, 5, 6, 7, 8, 9, 10, 11). For strains that could grow in liquid media (all except J193^T^ and J372^T^), solid media were replaced by liquid media of preferred composition with the same adjusted pH. Each experiment was carried out in two independent runs, each time in triplicates. Test results were evaluated 5 and 10 days after inoculation to determine if cells could grow under stress but with extended cultivation time. In order to examine anaerobic growth, inoculated medium plates of strain preference were cultivated under anerobic conditions using an anaerostat and AnaeroGen 2.5 l atmosphere generation system (ThermoFisher Scientific).

#### Enzymatic profile assessment and determination of substrate utilization capacity

The enzymatic profile was examined using the API ZYM kit (bioMérieux, France) according to the manufacturer’s instructions. Briefly, for each strain, bacterial suspension (5 McFarland units) was added to each strip well. The inoculated strips were incubated at the temperature preferred by the strain tested (28–37 °C). Cultivation was terminated by adding reaction solutions and subtracting the color change. Instead of the recommended 4 h, all tests were evaluated after an extended period of time (1–3 days, depending on the growth rate of the strains). The oxidase test was performed with OXItest (Erba Lachema) by immersion of a test strip in the freshly grown colony. The catalase test was carried out as a reaction of the fresh culture with 3%_v/v_ H_2_O_2_. Substrate assimilation was determined using a GEN III MicroPlate™ (Biolog, USA) according to the manufacturer’s instructions. For each strain, a bacterial suspension (0.5 McFarland units) was prepared by mixing the freshly grown bacterial culture and the chromogenic medium supplied with the kit. Inoculated 96-well plates were incubated at the preferred temperature of each strain. The evaluation of the test was carried out every 24 h for one week.

#### Fatty acid methyl ester (FAME) analysis

To determine the FAME profile, each strain was inoculated onto several R2A or 1/5 TSA plates according to their preference and cultured at each strain’s optimum temperature for 24–96 h depending on growth rate. Once the cultures were grown, cells were harvested from all plates, washed with 0.9% saline solution, and lyophilized until 100 mg of freeze-dried cells were harvested. Extraction and gas chromatography analysis of fatty acids were carried out as described elsewhere [49]. Briefly, the lyophilized biomass was saponified in 10% potassium hydroxide in methanol at laboratory temperature overnight. The obtained fatty acid fraction was divided into an alkalic solution (pH 9) and diethyl ether in order to remove basic and neutral components. The aqueous phase with fatty acids was acidified to pH 2 and extracted with hexane and then methylated using boron trifluoride in methanol [50, 51]. All obtained GC mass spectra were scanned within the *m*/*z* range of 50–600. By comparison of retention times and fragmentation patterns with the standards, the structures of the FAMEs were confirmed.

#### Declaration of generative AI and AI-assisted technologies in the manuscript preparation process

This article used artificial intelligence tools, such as DeepL and ChatGPT, to primarily correct the grammar and improve the style of the text. ChatGPT was also used to conduct a literature review and acquire the fatty acid compositions of the discussed bacteria. After applying these tools, the first and senior authors reviewed and edited the content as needed, taking full responsibility for the published article.

## Results and discussion

### The taxonomic classification of novel strains

Whole-genome sequencing and comprehensive genomic analyses were performed for all strains, providing detailed insights into their genomic characteristics. The sizes of the genomes ranged from 3.53 to 6.38 Mbp. The G + C content was > 70% in two strains, with a maximum value of 71.7% in strain J223^T^, and > 60% in five strains, while in one strain the G + C content was below 50% (J372^T^, 42.4%), as detailed in Supplementary table S1. At the level of phyla, these nine strains were classified into *Pseudomonadota* (*n* = 7) and *Bacteroidetes* (*n* = 2). The 16S rRNA gene similarity of 6/9 strains to the closest type strains cataloged in the RefSeq RNA database (NCBI) NCBI database and EZBioCloud 16S rRNA gene sequence database was lower than the suggested species delineation cut-off of 98.65% [52] (Table 1). However, strains J193^T^, J276^T^ and J452^T^ could be assigned to the recently described type species based on 16S rRNA gene similarity > 98.7%. Specifically, strain J193^T^ was related to *Hymenobacter guriensis* BT594^T^, strain J276^T^ to *Aquincola agrisoli* MAHUQ-54^T^ and strain J452^T^ to *Pseudomonas campi* S1-A32-2^T^. Nevertheless, the ANI values of all strains compared to their relatives, using either the GTDB database or NCBI type strains as references, were substantially lower than the suggested taxa delineation threshold of > 95% (Table 2). The only exception was the strain J276^T^, sharing an ANI of > 95% with *Aquincola tertiaricarbonis* MIMtkpLc11, a species that has not been validly published. Thus, strain J276^T^ still meets the above mentioned criterium as all other type strains. Furthermore, our analyses suggest that strain MIMtkpLc11 was misclassified as *Aquincola tertiaricarbonis*; even the authors of the original publication, which was a draft genome sequence announcement [53], acknowledged that the similarity between the 16S rRNA gene sequences of strains MIMtkpLc11 and *A. tertiaricarbonis* L10^T^ is only 98.36%. This value is below the threshold for assigning two strains to the same species. In fact, the similarity between the 16S rRNA gene sequences of strains MIMtkpLc11 and *Aquincola agrisoli* MAHUQ-54^T^ exceeds the reported value of 98.36%. In any case, our data indicate that strain MIMtkpLc11 should be classified in the same species as strain J276^T^, which is presented herein.

**Table 2:** The closest type strains based on the average nucleotide identity (ANI)

Strain	GTDB closest representative based on ANI calculated by Protologger	Hit (%)	NCBI closest type strain based on ANI	Hit (%)
J193	*Hymenobacter guriensis* BT594^T^	91.43	*Hymenobacter psychrotolerans* Tibet-IIU11^T^	78.13
J219	*Rhizobacter gummiphilus* NS21^T^	77.77	*Rhizobacter gummiphilus* NS21^T^	77.77
J223	*Caldimonas thermodepolymerans* K14^T^	81.29	*Caldimonas thermodepolymerans* K14^T^	81.29
J276	Aquincola tertiaricarbonis* MIMtkpLc11	98.68	*Aquincola agrisoli* MAHUQ-54^T^	84.79
J367	*Phenylobacterium kunshanense* BUT-10^T^	81.59	*Phenylobacterium kunshanense* BUT-10^T^	81.59
J372	*Flavobacterium johnsoniae* CI04	78.34	*Flavobacterium akiainvivens* IK-1^T^	77.82
J426	*Phenylobacterium kunshanense* BUT-10^T^	81.63	*Phenylobacterium kunshanense* BUT-10^T^	81.63
J428	*Mesorhizobium hawassense* AC99b^T^	80.07	*Mesorhizobium hawassense* AC99b^T^	80.07
J452	*Pseudomonas alcaligenes* MB-090714	86.43	*Pseudomonas campi* S1-A32-2^T^	93.41

*Suggested misclassification at the species level, see the main text for details

Phylogenetic and genomic analyses, including 16S rRNA sequencing, phylogeny reconstruction based on secondary structure of 16S rRNA gene (Supplementary figures S1-S3, S5, S7-S10), ANI, and POCP (Table 3), consistently indicate the taxonomic placement of the investigated strains. Specifically, strain J193^T^ was classified in the genus *Hymenobacter*, strains J367^T^ and J426^T^ in the genus *Phenylobacterium*, strain J372^T^ in the genus *Flavobacterium*, strain J428^T^ in the genus *Mesorhizobium* and strain J452^T^ in the genus *Pseudomonas*. Nonetheless, the core gene-based phylogenetic assessment revealed that all strains formed distinct branches from their related taxa (Supplementary figures S4 and S6 in this article; Figure S2 - Figure S11 published in Kapinusova et al. 2022 [5]). For strains J219^T^, J223^T^, J276^T^ where the taxonomic categorization based on ANI and POCP did not clearly place them in an existing genus within the order *Burkholderiales*, the AAI analysis was performed (Table 4). Based on the results of all analyses, the type strain most closely related to J219^T^ was classified as *Rhizobacter gummiphilus* NS21^T^, with 77.77% ANI, 58.97% POCP and 71.99% AAI. Thus, strain J219^T^ was assigned to the genus *Rhizobacter*. However, none of the analyses provided a clear picture of the taxonomic classification of strains J223^T^ and J276^T^. The affiliation of strain J223^T^ to the genus *Aquabacterium* was therefore mainly based on the 16S rRNA gene secondary structure (Supplementary figure S3) and the core gene-based phylogenetic tree (Supplementary figure S4), in which strain J223^T^ clustered with other members of the genus *Aquabacterium*. The same approach was applied to strain J276^T^, for which the 16S rRNA gene secondary structure (Supplementary figure S5) and the core gene-based phylogenetic tree (Supplementary figure S6) revealed clustering of J276^T^ with other species of the genus *Aquincola*.

**Table 3:** The closest type strains based on the percentage of conserved proteins (POCP)

Strain	GTDB closest representative based on POCP calculated by Protologger	Hit (%)	NCBI closest type strain based on POCP	Hit (%)
J193	*Hymenobacter psychrotolerans* Tibet-IIU11^T^	70.45	*Hymenobacter psychrotolerans* Tibet-IIU11^T^	70.45
J219	*Rhizobacter gummiphilus* NS21^T^	58.97	*Rhizobacter gummiphilus* NS21^T^	58.97
J223	Aquincola tertiaricarbonis* MIMtkpLc11	57.89	*Caldimonas thermodepolymerans* K14^T^	57.08
J276	Aquincola tertiaricarbonis* MIMtkpLc11	90.46	*Ideonella azotifigens* 1a22^T^	56.64
J367	*Phenylobacterium kunshanense* BUT-10^T^	62.18	*Phenylobacterium kunshanense* BUT-10^T^	62.18
J372	*Flavobacterium subsaxonicum* WB 4.1-42^T^	62.66	*Flavobacterium subsaxonicum* WB 4.1-42^T^	62.66
J426	*Phenylobacterium kunshanense* BUT-10^T^	65.23	*Phenylobacterium kunshanense* BUT-10^T^	65.23
J428	*Mesorhizobium qingshengii* CCBAU 33,460^T^	55.30	*Mesorhizobium qingshengii* CCBAU 33,460^T^	55.30
J452	*Pseudomonas alcaligenes* MB-090714	79.99	*Pseudomonas campi* S1-A32-2^T^	88.25

*Suggested misclassification at the species level, see the main text for details

**Table 4:** The closest type strains based on average amino acid identity (AAI)

Strain	Valid closest type strain based on AAI	Hit (%)
J219	*Rhizobacter gummiphilus* NS21^T^	71.99
J223	*Piscinibacter aquaticus* SH-1^T^	69.71
J276	*Aquincola agrisoli* MAHUQ-54^T^	80.59

### Physiology and chemotaxonomy

**Fig. 1:**
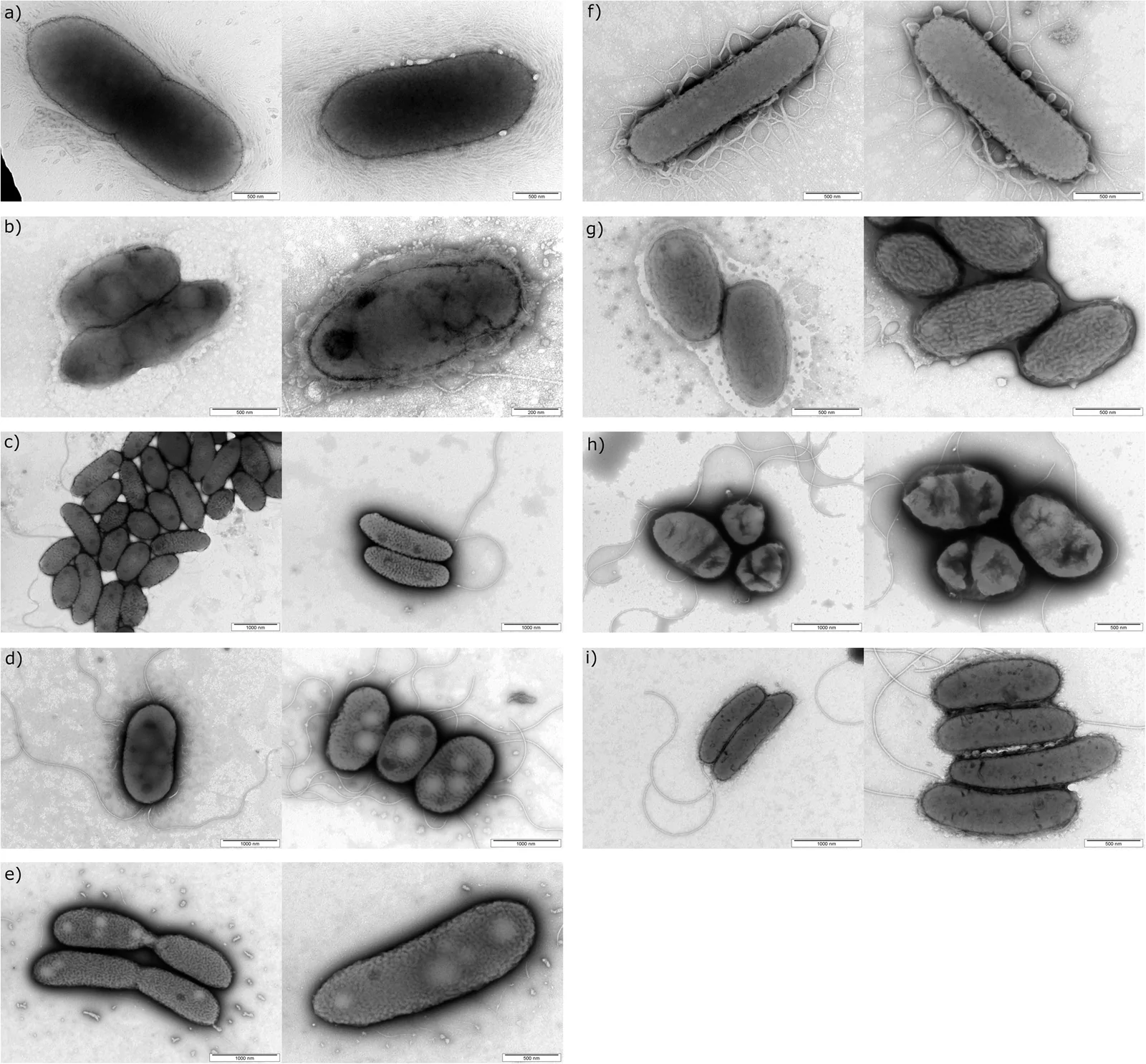
The cell morphology of non-motile strains examined using electron microscopy: **a** J193^T^; **b** J219^T^; **c** J223^T^; **d** J276^T^; **e** J367^T^; **f** J372^T^; **g** J426^T^; **h** J428^T^; **i** J452^T^

Strains J193^T^, J219^T^, J367^T^, J372^T^ and J426^T^ were non-motile rods, while strains J223^T^, J276^T^, J428^T^ and J452^T^ were motile rods or coccoid rods with single polar or multiple flagella (Fig. 1). The majority of the characterized strains were able to grow in liquid media. The preferred media were R2A and 1/5 Tryptic Soy Broth (TSB). For strains J219^T^ and J452^T^, LB medium was also suitable. However, no growth was observed for J193^T^ and J372^T^ in any of the tested liquid media, even when supplemented with solid supports such as sea sand, glass beads, or low-concentration agar [54, 55]. All strains thrived on solid media. Specifically, 1/10 PCA and 1/5 TSA were preferred by strains J193^T^, J372^T^ and J452^T^; 1/5 TSA by strains J367^T^ and J426^T^; and R2A by strains J219^T^, J223^T^, J276^T^, J428^T^. The temperature range at which each strain grew corresponded to the temperature of the hot springs in which the strains naturally occur: 20–32 °C for strains J193^T^ and J372^T^ and 20–37 °C for the other strains (Table 5). Compared with the closest type strains of the *Hymenobacter* genus, which are mainly isolated from cold deserts or glaciers [7, 9, 56], strain J193^T^ cannot thrive at temperatures as low as 4–10 °C (Supplementary table S2). Conversely, strain J219^T^ exhibited growth at 44 °C (Supplementary table S3). Strains J223^T^ and J276^T^ were found to have preferred mesophilic temperatures similar to other representatives of the genera *Aquabacterium* (Supplementary table S4) and *Aquincola* (Supplementary table S5). However, no match was found with representatives of the genus *Caldimonas* and *Calidifontimicrobium*, which prefer temperatures of 50 °C or higher [57, 58]. Finally, strain J426^T^ was able to grow at temperatures as low as 12 °C (Table 5, Supplementary table S6).

**Table 5:** Phenotypic characteristics and resistance abilities of novel Jáchymov strains. Symbols: +, positive; −, negative; w, weak activity/utilization

	J193^T^	J219^T^	J223^T^	J276^T^	J367^T^	J372^T^	J426^T^	J428^T^	J452^T^
Cell size (µm)	1.1 × 2.7	0.5 × 1-1.6	0.5 × 0.9–2.1	0.5 × 1.5	0.5 × 1.8	0.6 × 2	0.5 × 0.8	0.5 × 1	0.4 × 1.2
Motility	-	-	+	+	-	-	-	+	+
Flagella	none	none	single	single	none	none	none	multiple monopolar	single
Gram staining	negative	negative	negative	negative	negative	negative	negative	negative	negative
Colony color	pink	yellowish	transparent-white	white	white	yellow	white	white	white
O_2_ demand	aerobic	aerobic	aerobic	aerobic	aerobic	aerobic	aerobic	aerobic	aerobic
Growth range (optimum):									
Temperature (°C)	20–32 (30)	12–44 (37)	28–37 (32–37)	20–37 (24–28)	20–37 (30)	20–32 (30)	12–37 (30)	20–32 (30)	20–37 (32)
NaCl (%_w/v_)	≤ 0.5	≤ 3.5 (0.5-1)	≤ 4 (0.5-2)	≤ 1 (0.5)	≤ 1.5 (0.5)	≤ 0.5	≤ 2.5 (1)	0.5-2 (1)	0.5-2 (1)
pH	6–11	6.5–10	6–9 (8–9)	7–10 (8–9)	6–10 (8–9)	6–11	6–10 (7–8)	6–11	6–11
Basic enzymatic profile:									
Oxidase	-	+	+	+	-	-	-	-	+
Catalase	-	-	-	+	+	-	+	-	-
Alkaline phosphatase	+	+	w	w	+	+	+	+	w
Esterase (C4)	+	+	+	+	+	+	+	+	+
Esterase-lipase (C8)	+	+	+	+	w	+	+	+	+
Lipase (C14)	+	w	-	-	-	-	-	+	+
Leucine-arylamidase	+	+	+	+	+	+	+	+	+
Valine-arylamidase	+	-	-	+	+	+	+	-	-
Cystine-arylamidase	+	-	-	-	-	+	+	w	-
Trypsin	w	-	-	-	+	+	+	+	-
α-chymotrypsin	-	-	-	-	+	-	+	-	-
Acid phosphatase	+	-	-	w	+	-	+	+	+
Naphthol-AS-BI-phosphohydrolase	+	+	+	+	+	+	+	w	w
α-galactosidase	-	-	-	-	-	-	-	-	-
β-galactosidase	-	-	-	-	+	-	-	-	-
β-glucuronidase	-	-	-	-	-	+	-	-	-
α-glucosidase	+	+	-	+	+	+	+	+	-
β-glucosidase	w	-	-	-	-	w	-	-	-
N-acetyl-β-glucosaminidase	+	-	-	-	+	-	-	-	-
α-mannosidase	-	-	-	-	w	-	-	-	-
α-fucosidase	-	-	-	-	-	-	-	-	-
Assimilation of:									
Dextrin	-	-	-	+	-	+	-	-	-
d-Maltose	-	-	-	+	-	+	-	-	-
d-Trehalose	-	-	-	+	-	-	-	-	-
d-Cellobiose	-	-	-	+	-	-	-	+	-
Gentiobiose	-	-	-	+	-	+	-	+	-
Sucrose	-	-	-	+	-	-	-	-	-
d-Turanose	-	+	-	+	-	-	-	-	-
Stachyose	-	-	-	+	-	-	-	-	-
N-Acetyl-d-Glucosamine	-	-	-	+	-	-	-	-	-
α-d-Glucose	-	-	-	+	-	+	-	+	-
d-Mannose	-	-	-	+	-	+	-	+	-
d-Fructose	-	-	-	+	-	-	-	+	-
d-Galactose	-	-	-	+	-	-	-	+	-
d-Fucose	-	-	-	+	-	-	-	+	-
l-Rhamnose	-	-	-	+	-	-	-	-	-
d-Sorbitol	-	-	-	+	-	-	-	+	-
d-Mannitol	-	-	-	+	-	-	-	+	-
d-Arabitol	-	-	-	+	-	-	-	+	-
Glycerol	-	-	-	+	-	-	-	+	-
d-Glucose-6-PO_4_	-	+	-	-	-	-	-	-	-
d-Fructose-6-PO_4_	-	+	+	+	-	-	-	-	-
d-Aspartic Acid	-	-	-	+	-	-	-	-	-
d-Serine	-	-	-	-	-	-	-	-	-
Gelatin	-	-	-	-	-	+	+	-	-
Glycyl-l-Proline	-	-	-	-	+	-	+	-	-
l-Alanine	-	-	-	-	-	-	-	-	+
l-Arginine	-	-	-	-	-	-	-	-	+
l-Aspartic Acid	-	-	-	+	-	-	-	-	+
l-Glutamic Acid	-	-	-	+	-	+	+	-	+
l-Histidine	-	+	-	-	-	-	-	-	+
l-Pyroglutamic Acid	-	-	-	+	-	-	-	-	-
l-Serine	-	-	-	-	-	-	-	-	+
Pectin	-	-	-	+	-	-	-	-	-
D-Galacturonic Acid	-	-	-	-	-	+	-	+	-
l-Galactonic Acid Lactone	-	-	-	-	-	+	-	-	-
d-Gluconic Acid	+	-	-	+	-	-	-	-	-
d-Glucuronic Acid	-	+	-	-	-	-	-	+	-
Glucuronamide	+	+	-	-	-	-	-	-	-
Quinic Acid	-	-	-	+	-	-	-	+	-
d-Saccharic Acid	-	-	-	+	-	-	-	-	-
Methyl Pyruvate	-	-	-	-	-	-	-	-	+
d-Lactic Acid Methyl Ester	-	-	-	-	-	-	-	+	-
l-Lactic Acid	-	-	-	+	-	-	-	+	-
α-Ketoglutaric Acid	+	+	+	-	-	-	-	-	-
d-Malic Acid	-	+	-	+	-	-	-	-	-
l-Malic Acid	-	-	-	+	-	-	-	-	+
Bromosuccinic Acid	-	-	-	-	-	-	-	-	+
Tween 40	-	+	-	+	+	-	+	+	+
γ-Aminobutyric Acid	-	-	-	+	+	-	-	-	+
α-Hydroxybutyric Acid	-	+	-	-	-	-	-	-	-
β-Hydroxy-d,l-butyric Acid	-	+	-	+	-	-	+	-	+
α-Ketobutyric Acid	+	-	-	-	-	-	-	-	-
Acetoacetic Acid	+	+	+	+	+	+	+	+	-
Propionic Acid	-	-	-	-	-	-	-	-	+
Acetic Acid	-	-	-	+	+	+	-	-	+
Formic Acid	-	+	-	-	-	-	-	-	+
Resistance to antimicrobial compounds:									
1% Sodium Lactate	-	+	-	-	-	-	-	+	-
Lincomycin	-	+	+	-	+	-	-	+	+
Vancomycin	-	+	+	-	-	-	-	-	+
Nalidixic Acid	-	+	+	-	+	+	+	+	-
Aztreonam	-	+	+	+	+	+	+	-	-
Fusidic Acid	-	+	+	-	+	-	+	-	-
Rifamycin SV	-	+	+	-	+	+	+	+	+
Guanidine HCl	-	-	+	-	-	-	-	-	-
Tetrazolium Violet	-	+	+	-	-	+	-	+	-
Lithium Chloride	-	-	-	-	-	+	-	+	-
Sodium Butyrate	-	+	+	-	-	-	-	-	-
d-Serine	-	+	-	-	-	-	-	-	-
Minocycline	-	+	+	-	-	-	-	-	-
Tetrazolium Blue	-	+	+	-	-	-	-	+	-
Potassium Tellurite	-	+	-	-	-	-	-	+	-
Sodium Bromate	-	-	-	-	-	-	+	-	-

All of the novel Jáchymov strains exhibited the ability to grow within a wide pH range of 6–10 or 6–11. These findings are consistent with those of all the closest type strains, except for strain J372^T^, which showed greater resistance to higher pH values than its closest type representatives (Supplementary table S7). Only strains J219^T^ and J223^T^ exhibited resistance to the presence of NaCl in the medium at concentrations of ≤ 3.5%_w/v_ (Supplementary table S3) and ≤ 4%_w/v_ (Supplementary table S4), respectively. Strains J376^T^, J426^T^, J428^T^ and J452^T^ tolerated concentrations ranging from 1.5 to 2.5%_w/v_. The growth of strains J193^T^, J276^T^, and J372^T^ required the lowest NaCl concentration of ≤ 1%_w/v_. When these tolerances were compared with those of the closest type strains, it was found that strains J219^T^, J223^T^, J367^T^ and J426^T^ were the only ones that were more tolerant than their closest relatives (Supplementary table S3, S4, S6). Conversely, strain J428^T^ exhibited reduced tolerance to the presence of NaCl in comparison with other *Mesorhizobium* species (Supplementary table S8). No growth of any strain was observed under anaerobic conditions, even after a two-week cultivation period. Therefore, all of the novel strains characterized here are classified as aerobic.

### The utilization of various substrates and basic enzymatic profile determination

According to the results of the API ZYM test, all Jáchymov strains produce esterase (C4) and leucine arylamidase (Table 5, Supplementary tables S2-S9). A positive, or at least weakly positive, reaction was confirmed even for alkaline phosphatase, esterase-lipase (C8) and naphthol-AS-BI-phosphohydrolase. Conversely, none of the strains can produce α-galactosidase or α-fucosidase. Strain J193^T^ along with the closest *Hymenobacter* species were positive for alkaline phosphatase, leucin-arylamidase, valine-arylamidase, acid phosphatase and negative for α-chymotrypsin, β-galactosidase, β-glucuronidase, α-mannosidase and α-fucosidase. Strain J219^T^ corresponded with the closest type species of the genus *Rhizobacter* that exhibited positive reaction for esterase (C4), esterase-lipase (C8), leucin-arylamidase, naphtol-AS-BI-phosphohydrolase and negative for trypsin, α-galactosidase, β-glucuronidase. The absence of β-glucuronidase, α-glucosidase, β-glucosidase, N-acetyl‑β‑glucosaminidase, α-mannosidase and α-fucosidase indicated the taxonomic similarity between strain J223^T^ and representatives of *Sphaerotilaceae*, and *Comamonadaceae* (Supplementary table S4). Strain J276^T^ and its closest *Burkholderiaceae* and *Comamonadaceae* type strains were positive for oxidase, esterase (C4), esterase-lipase (C8), leucine-arylamidase and naphthol-AS-BI‑phosphohydrolase, but negative for β-galactosidase. Strains J367^T^ and J426^T^, similarly to their closest type *Phenylobacterium* species, produce alkaline phosphatase, but they cannot produce lipase (C14), α-galactosidase, β-glucuronidase or α-fucosidase. Strain J372^T^ showed a positive reaction to leucine-arylamidase, but negative for α-chymotrypsin and N-acetyl‑β‑glucosaminidase. The same was true for the closest *Flavobacterium* type species [27, 59, 60]. Even though the strain J452^T^ showed a narrow enzymatic profile, the positive reactions for esterase-lipase (C8), leucine-arylamidase, and negative reactions for α-chymotrypsin, α-galactosidase, β-galactosidase, β-glucuronidase, α-glucosidase, β-glucosidase, N-acetyl‑β‑glucosaminidase, α-mannosidase and α-fucosidase were consistent with the enzymatic profile of the closest *Pseudomonas* type species [14, 61, 62]. Only the strain J428^T^ did not produce an enzyme that corresponded with those produced by *Mesorhizobium* species. This difference is most likely due to the different isolation sources, since other *Mesorhizobium* species are known to be associated with roots rather than being water-borne bacteria [11, 13, 63].

Substrate assimilation and basic sensitivity test to antimicrobial compounds, as determined using a GEN III MicroPlate™, showed that all strains except J276^T^ had the common ability to utilize only a narrow spectrum of substrates (Table 5). Specifically, strain J193^T^ was able to utilize only d-gluconic acid, glucuronamide, α-ketoglutaric acid, α-ketobutyric acid, acetoacetic acid, and was sensitive to all the antimicrobial compounds tested. Strain J223^T^ also expressed poor metabolic activity, utilizing only d-fructose-6-PO_4_, α-ketoglutaric and acetoacetic acid. On the contrary, strain J276^T^ exhibited a broad range of metabolic activities, utilizing the majority of the tested substrates. Strains J219^T^ and J223^T^ showed great resistance to a variety of antimicrobial compounds. Strain J219^T^ was only sensitive to guanidine HCl, lithium chloride, and sodium bromate, whereas strain J223^T^ was sensitive to 1% sodium lactate, lithium chloride, d-serine, potassium tellurite, and sodium bromate. For detailed information on the substrate assimilation ability of the Jáchymov strains and their closest type strains, refer to Supplementary tables S2–S9.

### Fatty acid profile

Analysis of the fatty acid profile revealed a feature common to all the novel Jáchymov strains: the presence of palmitic acid (C_16:0_, Supplementary table S10). The highest concentrations were found in strain J193^T^, where it was also the most dominant acid, accounting for 52.8% of the total, followed by J223^T^, where it appeared as the second most abundant acid with 37.4% (Supplementary table S10). The concentration of palmitic acid was also high in other strains, namely J276^T^ (29.4%), J219^T^ (25.4%) and J367^T^ (20.8%), while in strains J426^T^, J428^T^ and J452^T^ ranged between 13.5% and 16.6%. The only exception was strain J372^T^, for which C_16:0_ represented just 4.1% of the total profile. The significant presence of C_16:0_ in the Jáchymov strains can be explained by the fact that some bacteria use sphingolipids synthesized from palmitic acid to resist the oxidative stress [64–67], which is typical of Jáchymov radon waters.

Another most frequently found fatty acid in the Jáchymov strains, with the exception of strain J426^T^, was C_16:1_ ω7c, which had a concentration ranging from 1.5% to 39.1%. In strains J367^T^ and J426^T^, C_18:1_ ω7c was the most abundant fatty acid, reaching 72.4% and 64.2% of the total, respectively. Together with C_16:0_, which was also an abundant fatty acid, this phenomenon proved to be typical of the genus *Phenylobacterium* [68, 69]. The major fatty acid in J428^T^ was also C_18:1_ ω7c, and its high representation is also typical of other species of the genus *Mesorhizobium* [70–72]. Although the fatty acid composition of *Pseudomonas* species varies greatly, the dominance of C_18:1_ ω7c (42.5%) in strain J452^T^ was consistent with those of *Pseudomonas flavocrustae* with 46.6% [73] and *Pseudomonas glareae* with 36.5% [74]. Strain J372^T^ had the most distinct profile compared to all Jáchymov strains. However, its profile corresponded to those of other *Flavobacterium* strains. For example, iso-C_15:0_ was the most abundant fatty acid in both J372^T^ (47.9%) and *Flavobacterium okayamense* [75]. Anteiso-C_15:0_, found in strain J372^T^ at a concentration of 10.1%, was one of the major fatty acids in *Flavobacterium soyangense* [76]. The presence of fatty acids cyclo-C_17:0_, C_16:0_, C_18:1_ ω7c and C_16:1_ ω7c in strain J219^T^ corresponded to the fatty acid profile of other *Rhizobacterium* species [17, 77, 78]. In addition, the profiles of strain J193^T^ and other strains of the genus *Hymenobacter* showed a clear matching pattern when C_16:0_ and C_16:1_ ω7c were present. The taxonomic classification of J276^T^ to the genus *Aquincola* was supported by the presence of C_16:1_ ω7c as a major fatty acid in this strain and in the species *A. rivuli* [79] and *A. amnicola* [80]. Finally, the fatty acid profile of strain J223^T^, dominated by C_18:0_, differed from other *Aquabacterium* members, where this fatty acid was either completely absent or present in only small percentages.

### Final categorization of Jáchymov novel strains to the existing genera

Based on the results of the 16S rRNA gene phylogeny reconstructions, ANI, and POCP similarities to the closest type strains, here we propose strain J193^T^ as a novel member of the *Hymenobacter* genus; strain J372^T^ as a novel member of the *Flavobacterium* genus; strain J367^T^ together with J426^T^ as novel members of the *Phenylobacterium* genus; strain J428^T^ as a novel member of the *Mesorhizobium* genus; and strain J452^T^ as a novel member of *Pseudomonas* genus.

According to ANI, POCP, and AAI similarities, as well as the main chemotaxonomic features (such as the basic enzymatic profile and fatty acid composition), here we propose strain J219^T^ as a novel member of genus *Rhizobacter*.

According to 16S rRNA gene and core genome phylogeny reconstructions, as well as physiological and enzymatic profiling, we propose that strain J223^T^ is a novel member of the *Aquabacterium* genus.

Finally, as indicated by the 16S rRNA gene and core-genome phylogeny reconstructions, POCP and AAI similarities, together with chemotaxonomic features of enzymatic profile and fatty acid composition, here we propose strain J276^T^ as novel member of the *Aquincola* genus.

### Description of *Hymenobacter abyssi* sp. nov

*Hymenobacter abyssi* (a.bys’si. L. gen. n. *abyssi*, from the depth). Cells are Gram-stain-negative, non-spore-forming, non-motile, 1.1 μm wide and 2.7 μm long rods (Fig. 1a). After 5 days of cultivation on 1/5 TSA or 1/10 PCA medium, cells form 4–6 mm large, mucoid, pink colonies. No growth is observed in liquid media. Growth occurs in a temperature range of 20–32 °C with optimum 30 °C, on media with pH 6–11, and in a presence of ≤ 0.5%_w/v_ NaCl. The bacterium is oxidase and catalase negative. In the API ZYM test it is positive for alkaline phosphatase, esterase (C4), esterase lipase (C8), lipase (C14), leucin-arylamidase, valine-arylamidase, cystine-arylamidase, acid phosphatase, naphthol-AS-BI-phosphohydrolase, α-glucosidase and N-acetyl-β-glucosaminidase activity, but negative for α-chymotrypsin, α-galactosidase, β-galactosidase, β-glucuronidase, α-mannosidase and α-fucosidase. Weak activity was observed for trypsin and β-glucosidase. The utilized substrates are d-gluconic acid, glucuronamide, α-ketoglutaric acid, α-ketobutyric acid and acetoacetic acid. C_16:0_ (52.8%) and C_16:1_ ω7c (39.1%) acids are the major fatty acids. The genome size is 5.664 Mbp, with a DNA G + C content of 59.96%. The type strain is J193^T^ (= DSM 113871^T^ =CCM 9301^T^).

### Description of *Rhizobacter abyssi* sp. nov

*Rhizobacter abyssi* (a.bys’si. L. gen. n. *abyssi*, from the depth). Cells are Gram-stain-negative, non-spore-forming, non-motile and 0.5 μm wide x 1–1.6 μm long rods (Fig. 1b). Colonies grown on R2A medium are after 2 days of cultivation yellowish, circular, and 1–2 mm in diameter. Growth occurs in a temperature range of 12–44 °C with optimum 37 °C, on media with pH 6.5–10, and in a presence of ≤ 3.5%_w/v_ (optimum 0.5-1%_w/v_) NaCl. The bacterium is oxidase positive and catalase negative. In the API ZYM test it is positive for alkaline phosphatase, esterase (C4), esterase lipase (C8), leucin-arylamidase, naphthol-AS-BI-phosphohydrolase, α-glucosidase, but negative for valine-arylamidase, cystine-arylamidase, trypsin, α-chymotrypsin, acid phosphatase, α-galactosidase, β-galactosidase, β-glucuronidase, β-glucosidase, N-acetyl-β-glucosaminidase, α-mannosidase and α-fucosidase activity. Weak activity was observed for lipase (C14). Using Biolog GEN III test, the bacterium assimilates d-turanose acid, d-glucose-6-PO_4_, d-fructose-6-PO_4_, l-histidine, d-glucoronic acid, glucuronamide, α-keto-glutaric acid, d-malic acid, tween 40, α-hydroxybutyric acid, β-hydroxy-d,l-butyric acid, acetoacetic acid and formic acid. The bacterium is resistant to 1% sodium lactate, lincomycin, vancomycin, nalidixic acid, aztreonam, fusidic acid, rifamycin SV, tetrazolium violet, sodium butyrate, d-serine, minocycline, tetrazolium blue, potassium tellurite. Cyclo-C_17:0_ (32.6%), C_16:0_ (25.4%), C_18:1_ ω7c (16.7%) and C_16:1_ ω7c (16.5%) acids are the major fatty acids. The genome size is 5.524 Mbp, with a DNA G + C content of 67.70%. The type strain is J219^T^ (= DSM 113781^T^ =CCM 9293^T^).

### Description of *Aquabacterium abyssi* sp. nov

*Aquabacterium abyssi* (a.bys’si. L. gen. n. *abyssi*, from the depth). Cells are Gram-stain-negative, rod shaped, non-spore-forming and motile by means of single-polar flagellum. Cells are 0.5 × 0.9–2.1 μm in size (Fig. 1c). Colonies grown on R2A medium are after 7 days of cultivation transparent, circular, flat and ~ 1 mm in size. The bacterium is able to grow in a temperature range of 28–37 °C with optimum 32–37 °C, on media with pH 6–9 (optimum 8–9), and in a presence of ≤ 4%_w/v_ (optimum 0.5-2%_w/v_) NaCl. It is positive for oxidase and negative for catalase activities. In the API ZYM test reactions are positive for esterase (C4), esterase lipase (C8), leucin-arylamidase, naphthol-AS-BI-phosphohydrolase, but negative for lipase (C14), valine-arylamidase, cystine-arylamidase, trypsin, α-chymotrypsin, acid phosphatase, α-galactosidase, β-galactosidase, β-glucuronidase, α-glucosidase, β-glucosidase, N-acetyl-β-glucosaminidase, α-mannosidase and α-fucosidase activity. Weak activity was observed for alkaline phosphatase. In the Biolog Gen III test, reactions are positive for utilization of d-fructose-6-PO_4_, α-ketoglutaric acid, acetoacetic acid and the bacterium is resistant to lincomycin, vancomycin, nalidixic acid, aztreonam, fusidic acid, rifamycin SV, guanidine HCl, tetrazolium violet, sodium butyrate, minocycline and tetrazolium blue. C_18:0_ (39.6%), C_16:0_ (37.4%), cyclo-C_17:0_ (17.3%) acids are the major fatty acids. The genome size is 4.656 Mbp, with a DNA G + C content of 71.73%. The type strain is J223^T^ (= DSM 113847^T^ =CCM 9303^T^).

### Description of *Aquincola abyssi* sp. nov

*Aquincola abyssi* (a.bys’si. L. gen. n. *abyssi*, from the depth). Cells are Gram-stain-negative, rod shaped, non-spore-forming and motile by a single polar flagellum. Cells are 0.5 × 1.5 μm in size (Fig. 1d). Colonies grown on R2A medium are after 24 h of cultivation white, irregular, ingrown to agar and ~ 2 mm in size. Growth occurs in a temperature range of 20–37 °C with optimum 24–28 °C, on media with pH 7–10 (optimum 8–9), and in a presence of ≤ 1%_w/v_ (optimum 0.5%_w/v_) NaCl. Positive for oxidase and catalase activities. In the API ZYM test reactions are positive for esterase (C4), esterase lipase (C8), leucin-arylamidase, valine-arylamidase, α-glucosidase, but negative for lipase (C14), cystine-arylamidase, trypsin, α-chymotrypsin, α-galactosidase, β-galactosidase, β-glucuronidase, β-glucosidase, naphthol-AS-BI-phosphohydrolase, N-acetyl-β-glucosaminidase, α-mannosidase and α-fucosidase activity. Weak activity was observed for alkaline phosphatase and acid phosphatase. The bacterium assimilates dextrin, d-maltose, d-trehalose, d-cellobiose, gentiobiose, sucrose, d-turanose, stachyose, N-acetyl-d-glucosamine, α-d-glucose, d-mannose, d-fructose, d-galactose, d-fucose, l-rhamnose, d-sorbitol, d-mannitol, d-arabitol, glycerol, d-fructose-6-PO_4_, d-aspartic acid, l-aspartic acid, l-glutamic acid, l-pyroglutamic acid, pectin, d-gluconic acid, quinic acid, d-saccharic acid, l-lactic acid, d-malic acid, l-malic acid, tween 40, γ-aminobutyric acid, β-hydroxy-d,l-butyric acid, acetoacetic acid and acetic acid. Resistant to aztreonam. Cyclo-C_17:0_ (31.7%), C_16:0_ (29.4%), C_16:1_ ω7c (14.3%) acids are the major fatty acids. The genome size is 6.375 Mbp, with a DNA G + C content of 70.12%. The type strain is J276^T^ (= DSM 113828^T^ =CCM 9294^T^).

### Description of *Phenylobacterium abyssi* sp. nov

*Phenylobacterium abyssi* (a.bys’si. L. gen. n. *abyssi*, from the depth). Cells are Gram-stain-negative, non-spore-forming, non-motile rods with 0.5 × 1.8 μm in size (Fig. 1e). Colonies grown on 1/5 TSA medium are after 48 h of cultivation white, circular, convex and ~ 1 mm in size. Growth occurs in a temperature range of 20–37 °C with optimum 30 °C, on media with pH 6–10 (optimum 8–9), and in a presence of ≤ 1.5%_w/v_ (optimum 0.5%_w/v_) NaCl. Negative for oxidase and positive for catalase activities. According to API ZYM test, positive reactions are for alkaline phosphatase, esterase (C4), leucin-arylamidase, valine-arylamidase, trypsin, α-chymotrypsin, acid phosphatase, naphthol-AS-BI-phosphohydrolase, β-galactosidase, α-glucosidase, N-acetyl-β-glucosaminidase, but negative for lipase (C14), cystine-arylamidase, α-galactosidase, β-glucuronidase, β-glucosidase and α-fucosidase. Weak activity was observed for esterase lipase (C8) and α-mannosidase. In the Biolog Gen III test bacterium assimilates glycyl-l-proline, tween 40, γ-aminobutyric acid, acetoacetic acid, acetic acid and is resistant to lincomycin, nalidixic acid, aztreonam, fusidic acid and rifamycin SV. C_18:1_ ω7c (72.4%) and C_16:0_ (20.8%) acids are the major fatty acids. The genome size is 4.954 Mbp, with a DNA G + C content of 69.96%. The type strain is J367^T^ (= DSM 113838^T^ =CCM 9302^T^).

### Description of *Flavobacterium abyssi* sp. nov

*Flavobacterium abyssi* (a.bys’si. L. gen. n. *abyssi*, from the depth). Cells are Gram-stain-negative, non-spore-forming, non-motile, 0.6 μm wide and 2 μm long rods (Fig. 1f). Colonies growing on 1/10 PCA medium are after 5 days of cultivation yellow, 3–5 mm large and mucoid. No growth is observed in liquid media. Able to grow at 20–32 °C (optimum, 30 °C), at pH 6–11 and in 0.5%_w/v_ NaCl. Negative for oxidase and catalase activities. According to API ZYM test results, positive reactions are for alkaline phosphatase, esterase (C4), esterase lipase (C8), leucin-arylamidase, valine-arylamidase, cystine-arylamidase, trypsin, naphthol-AS-BI-phosphohydrolase, β-glucuronidase, α-glucosidase, but negative for lipase (C14), α-chymotrypsin, acid phosphatase, α-galactosidase, β-galactosidase, N-acetyl-β-glucosaminidase, α-mannosidase, and α-fucosidase. Weak activity was observed for β-glucosidase. In the Biolog Gen III test, the bacterium assimilates dextrin, d-maltose, gentiobiose, α-d-glucose, d-mannose, gelatin, l-glutamic acid, d-galacturonic acid, acetoacetic acid, acetic acid and is resistant to nalidixic acid, aztreonam, rifamycin SV, tetrazolium violet and lithium chloride. Iso-C_15:0_ (47.9%), iso-C_16:0_ (13.6%) and anteiso-C_15:0_ (10.1%) acids are the major fatty acids. The genome size is 3.53 Mbp, with a DNA G + C content of 42.42%. The type strain is J372^T^ (= DSM 113872^T^ =CCM 9299^T^).

### Description of *Phenylobacterium fontinale *sp. nov

*Phenylobacterium fontinale (fon.ti.na’le. L. neut. adj. fontinale*,* of a spring)*. Cells are Gram-stain-negative, non-spore-forming, non-motile, 0.5 μm wide and 0.8 μm long coccoid rods (Fig. 1g). Colonies grown on 1/5 TSA medium are after 48 h of cultivation white, raised and ~ 1 mm in size. Growth occurs at 12–37 °C (optimum 30 °C), at pH 6–10 (optimum 8–9) and in a presence of ≤ 2.5%_w/v_ (optimum 1%_w/v_) NaCl. Negative for oxidase and positive for catalase activities. In the API ZYM test are positive reactions for alkaline phosphatase, esterase (C4), esterase lipase (C8), leucin-arylamidase, valine-arylamidase, cystine-arylamidase, trypsin, α-chymotrypsin, acid phosphatase, naphthol-AS-BI-phosphohydrolase, α-glucosidase, but negative for lipase (C14), α-galactosidase, β-galactosidase, β-glucuronidase, β-glucosidase, N-acetyl-β-glucosaminidase, α-mannosidase, and α-fucosidase. Using Biolog GEN III test, bacterium assimilates gelatin, glycyl-l-proline, l-glutamic acid, tween 40, β-hydroxy-d,l-butyric acid, acetoacetic acid and is resistant to nalidixic acid, aztreonam, fusidic acid, rifamycin SV and sodium bromate. C_18:1_ ω7c (64.2%) and C_16:0_ (13.5%) acids are the major fatty acids. The genome size is 4.64 Mbp, with a DNA G + C content of 69.04%. The type strain is J426^T^ (= DSM 113839^T^ =CCM 9296^T^).

### Description of *Mesorhizobium abyssi* sp. nov

*Mesorhizobium abyssi* (a.bys’si. L. gen. n. *abyssi*, from the depth). Cells are Gram-stain-negative, non-spore-forming, motile by polar multiple flagella. Cell size is 0.5 × 1 μm (Fig. 1h). After 3 days of cultivation on R2A medium, cells form ~ 2 mm large, white, flat colonies. Growth occurs in a temperature range of 20–32 °C with optimum 30 °C, on media with pH 6–11 (optimum 8–9), and in a presence of ≤ 0.5-2%_w/v_ NaCl (optimum 1%_w/v_). The bacterium is oxidase and catalase negative. In the API ZYM test, it is positive for alkaline phosphatase, esterase (C4), esterase lipase (C8), lipase (C14), leucin-arylamidase, trypsin, acid phosphatase, α-glucosidase, but negative for valine-arylamidase, α-chymotrypsin, α-galactosidase, β-galactosidase, β-glucuronidase, β-glucosidase, N-acetyl-β-glucosaminidase activity α-mannosidase and α-fucosidase. Weak activity was observed for cystine-arylamidase and naphthol-AS-BI-phosphohydrolase. The utilized substrates are d-cellobiose, gentiobiose, α-d-glucose, d-mannose, d-fructose, d-galactose, d-fucose, d-sorbitol, d-mannitol, d-arabitol, glycerol, d-galacturonic acid, d-glucuronic acid, quinic acid, d-lactic acid methyl ester, l-lactic acid, tween 40 and acetoacetic acid. The bacterium is resistant to 1% sodium lactate, lincomycin, nalidixic acid, rifamycin SV, tetrazolium violet, lithium chloride, tetrazolium blue and potassium tellurite. C_18:1_ ω7c (55.3%), C_16:0_ (13.9%) and C_18:0_ (11.2%) acids are the major fatty acids. The genome size is 5.882 Mbp, with a DNA G + C content of 65.05%. The type strain is J428^T^ (= DSM 114114^T^ =CCM 9297^T^).

### Description of *Pseudomonas fontinalis* sp. nov

*Pseudomonas fontinalis* (fon.ti.na’lis. L. fem. adj. *fontinalis*, of a spring). Cells are Gram-stain-negative, non-spore-forming, motile by single polar flagellum and 0.4 μm wide x 1.2 μm long rods (Fig. 1i). Colonies grown on 1/10 PCA medium are after 24 h of cultivation white, circular, and 1–2 mm in size. Growth occurs in a temperature range of 20–37 °C with optimum 32 °C, on media with pH 6–10 (optimum 7–8), and in a presence of 0.5-2%_w/v_ (optimum 1%_w/v_) NaCl. The bacterium is oxidase positive and catalase negative. In the API ZYM test, it is positive for esterase (C4), esterase lipase (C8), lipase (C14), leucin-arylamidase, acid phosphatase, but negative for valine-arylamidase, cystine-arylamidase, trypsin, α-chymotrypsin, α-galactosidase, β-galactosidase, β-glucuronidase, α-glucosidase, β-glucosidase, N-acetyl-β-glucosaminidase, α-mannosidase and α-fucosidase activity. Weak activity was observed for alkaline phosphatase and naphthol-AS-BI-phosphohydrolase. In Biolog GEN III test, the bacterium assimilates l-alanine, l-arginine, l-aspartic acid, l-glutamic acid, l-histidine, l-serine, methyl pyruvate, l-malic acid, bromo-succinic acid, tween 40, γ-aminobutyric acid, β-hydroxy-d,l-butyric acid, propionic acid, acetic acid and formic acid. The bacterium is resistant to lincomycin, vancomycin and rifamycin SV. C_18:1_ ω7c (42.5%), C_16:1_ ω7c (30.7%), and C_16:0_ (16.6%) acids are the major fatty acids. The genome size is 4.863 Mbp, with a DNA G + C content of 63.21%. The type strain is J452^T^ (= DSM 113783^T^ =CCM 9298^T^).

## Supplementary Information

Supplementary Material 1.

## Data Availability

The genome assemblies of the strains are available at the NCBI Genome Archive under BioProject ID PRJNA782494.The accession numbers for the whole-genome sequences of the strains are as follows: J193—NZ_JAJONE000000000, J219—NZ_JAJOND000000000, J223—NZ_CP088297, J276—NZ_JAJONC000000000, J367—NZ_JAJONA000000000, J372—NZ_JAJOMZ000000000, J426—NZ_JAJOMY000000000, J428—NZ_JAJOMX000000000, J452—NZ_CP088294. The GenBank accession numbers for the 16S rRNA gene sequences of the strains are as follows: J193—PX529875, J219—PX529876, J223—PX529877, J276—PX529878, J367—PX529879, J372—PX529880, J426—PX529881¸ J428—PX529882¸ J452—PX529883.

## Abbreviations

ortho ANI: orthologous average nucleotide identity
AAI: average amino acid identity
POCP: percentage of conserved proteins
UBCG: up-to-date bacterial core gene
