# Supplementary material for: Beyond the boundaries of microbial diversity in radioactive environments: novel microbial species isolated from a former silver-uranium mine’s radon-saturated waters
Source: BMC Microbiol. 2026 Jul 13;26:889. doi: 10.1186/s12866-026-05379-9 (PMC13670492; doi:10.1186/s12866-026-05379-9)
Supplement: Supplementary file 1 [file 12866_2026_5379_MOESM1_ESM.pdf]

Beyond the boundaries of microbial diversity in radioactive environments: novel microbial species isolated from a former silver-uranium mine's radon-saturated waters

Gabriela Kapinusova[1], Kunal Jani[2], Petr Pajer[3], Alena Filipova[4], Pavel Ulbrich[1], Tomas Cajthaml[4], Michal Strejcek[1], Jachym Suman[1], Ondrej Uhlik[1]

[1] University of Chemistry and Technology, Prague, Faculty of Food and Biochemical Technology, Department of Biochemistry and Microbiology, Prague, Czech Republic

[2] Universität Ulm, Institute of Evolutionary Ecology and Conservation Genomics, Ulm, Germany

[3] Military Health Institute, Ministry of Defence of the Czech Republic, Prague, Czech Republic

[4] Institute of Microbiology of the Czech Academy of Sciences, Prague, Czech Republic

*Correspondence: Gabriela Kapinusova, gabriela.kapinusova@vscht.cz; Ondrej Uhlik, ondrej.uhlik@vscht.cz

Supplementary table S1: Genome sequencing summary

	***Hymenobacter abyssi* J193^T**	***Rhizobacter abyssi* J219^T**	***Aquabacterium abyssi* J223^T**	***Aquincola abyssi* J276^T**	***Phenylobacterium abyssi* J367^T**	***Flavobacterium abyssi* J372^T**	***Phenylobacterium fontinale* J426^T**	***Mesorhizobium abyssi* J428^T**	***Pseudomonas fontinalis* J452^T**
**Number of reads**	231241	163478	43503	86891	21930	62901	23661	159251	245813
**Longest read length (bp)**	4865854	5500031	4656067	3816059	4809436	3323599	4121349	5315803	4862927
**Average read length (bp)**	809165	2761985	4656067	3187657	2476961.5	504339.2857	1546543	1176435.2	4862927
**Contigs**	7	2	1	2	2	7	3	5	1
**Completeness (%)**	99.4	98.12	99.18	98.91	90.17	98.39	95.96	97.06	98.05
**Contamination (%)**	2.53	2.34	0.86	2.02	1.3	6.01	1.14	0.82	1.5
**Genome size (bp)**	5664156	5523971	4656067	6375314	4953923	3530375	4639629	5882176	4862927
**GC content (%)**	59.96	67.70	71.73	70.12	69.96	42.42	69.04	65.05	63.21

Supplementary table S2: Comparison of phenotypic characteristics of strain J193^T and its closest type strains of the family *Hymenobacteraceae*. Strains: 1) J193^T; 2) *Hymenobacter guriensis* BT594^T (1); 3) *Hymenobacter lutimineralis* KIGAM108^T (2); 4) *Hymenobacter psychrotolerans* Tibet-IIU11^T (3); 5) *Hymenobacter glacieicola* B1909^T (4); 6) *Hymenobacter roseosalivarius* AA-718^T (5). Data for the reference strains were obtained from the available literature. All strains were Gram-stain negative. All *Hymenobacter* species in this comparison (except *H. roseosalivarius* AA-718^T where data are not available) were positive for alkaline phosphatase, leucin-arylamidase, valine-arylamidase, acid phosphatase and negative for α-chymotrypsin, β-galactosidase, β-glucuronidase, α-mannosidase and α-fucosidase. +, Positive; −, negative; w, weak activity/utilization; NA, not analyzed or not determined.

	1	2	3	4	5	6
Isolation source	mineral water	soil	zeolith	permafrost sediment	glacial ice core	sandstone
Cell size (µm)	1.1 x 2.7	0.5-1.2 x 3.5-4.3	1.2-1.4 x 3.2-4.9	0.5 x 0.8-1.7	1.1 x 3.5	1-1.25 x 2-2.7
Flagella	none	none	none	none	none	none
Colony color	pink	red	pink-red	light-pink	pink-red	pink-red
DNA G+C content (mol%)	59.96	59.8	60.0	60.0	59.0	56.0
**<u>Growth range (optimum):</u>**						
Temperature (°C)	20-32 (30)	10-30 (25)	4-37 (20-25)	4-28 (18)	4-30 (10)	3-25 (19-22)
NaCl ($\%_{w/v}$)	≤0.5	NA	≤0.5 (0)	≤1.5 (0)	0	0.5-1
pH	6-11	6-9 (8)	6-10 (7-8)	5-10 (7)	6-9 (6)	5-10.5 (7.5-8)
**<u>Basic enzymatic profile:</u>**						
Oxidase	-	+	+	+	+	+
Catalase	-	+	+	+	+	+
Esterase (C4)	+	w	+	+	+	NA
Esterase-lipase (C8)	+	w	+	+	+	NA
Lipase (C14)	+	-	-	-	-	-
Cystine-arylamidase	+	w	+	+	+	NA
Trypsin	w	-	+	-	+	NA
Naphthol-AS-BI-phosphohydrolase	+	w	+	+	+	NA
α-galactosidase	-	-	-	-	+	+
α-glucosidase	+	w	+	-	+	+

	1	2	3	4	5	6
β-glucosidase	w	w	-	-	+	+
N-acetyl-β-glucosaminidase	+	+	-	+	+	NA
**Assimilation of:**						
D-Maltose	-	-	-	-	NA	-
D-Trehalose	-	NA	-	-	NA	-
D-Cellobiose	-	NA	NA	-	NA	w
Sucrose	-	NA	-	+	NA	-
N-Acetyl-D-glucosamine	-	-	-	-	NA	-
α-D-Glucose	-	-	-	+	NA	w
D-Mannose	-	-	-	+	NA	w
D-Fructose	-	NA	-	NA	NA	-
D-Galactose	-	NA	-	NA	NA	-
L-Rhamnose	-	-	-	-	NA	-
D-Sorbitol	-	NA	-	NA	NA	+
D-Mannitol	-	NA	-	-	NA	+
Gelatin	-	NA	-	NA	NA	-
L-Alanine	-	NA	-	-	NA	-
L-Aspartic Acid	-	NA	NA	NA	NA	+
L-Glutamic Acid	-	NA	+	NA	NA	-
Pectin	-	NA	NA	NA	NA	-
Methyl Pyruvate	-	NA	+	-	NA	w

Supplementary table S3: Comparison of phenotypic characteristics of strain J219^T and its closest type strains of the family *Burkholderiaceae* and *Sphaerotilaceae*. Strains: 1) J219^T; 2) *Rhizobacter profundi* DS48-6-5^T (6); 3) *Rhizobacter gummiphilus* NS21^T (7); 4) *Rhizobacter bergeniae* PLGR-1^T (8); 5) *Rhizobacter dauci* H6^T (9); *Piscinibacter caeni* MQ-18^T (10). Data for the reference strains were obtained from the available literature. All strains were Gram-stain negative. All strains were oxidase positive. All *Rhizobacter* species in this comparison (except *R. gummiphilus* NS21^T where data are not available) were positive for esterase (C4), esterase-lipase (C8), leucin-arylamidase, naphtol-AS-BI-phosphohydrolase and negative for trypsin, α-galactosidase, β-glucuronidase. +, Positive; −, negative; w, weak activity/utilization; NA, not analyzed or not determined.

	1	2	3	4	5	6
Isolation source	mineral water	freshwater sediment	soil	root	soil/root	activated sludge
Cell size (µm)	0.47-0.53 x 1-1.6	NA	0.6-0.7 × 1.2-1.5	0.4–0.6 × 1.7–2.2	0.9-1.3 × 2.1-2.5	0.5–0.75 × 1.9–2.7
Motility	-	+	+	+	+	+
Flagella	none	single	single	single	multi-flagellar	single
Colony color	white-yellowish	brownish	yellowish	yellowish	white-yellowish	yellowish
DNA G+C content (mol%)	67.7	66.7	70.8	69.8	66.9-70.6	70.1
**Growth range (optimum):**						
Temperature (°C)	12-44 (37)	8-30	10-35	7-33 (25-28)	28-30	20-40 (30)
NaCl ($\%_{w/v}$)	≤3.5 (0.5-1)	>1	NA	≤0.5 (0)	≤0.5 (0)	≤0.8 (0)
pH	6.5-10	6-9 (7)	6-9	5-10 (6-7)	5-9	6-9 (7)
**Basic enzymatic profile:**						
Nitrate reduction	-	+	+	-	+	+
Catalase	-	-	+	+	+	+
Alkaline phosphatase	+	-	NA	+	+	-
Lipase (C14)	w	-	NA	-	-	-
Valine-arylamidase	-	-	NA	+	-	-
Cystine-arylamidase	-	-	NA	-	+	-
α-chymotrypsin	-	-	NA	+	-	-
Acid phosphatase	-	-	NA	+	-	+

	1	2	3	4	5	6
β-galactosidase	-	-	+	-	-	+
α-glucosidase	+	-	NA	+	-	+
β-glucosidase	-	-	NA	-	+	+
**Assimilation of:**						
D-Gluconic acid	+	-	+	-	-	+
D-Glucosa-6-PO_4	+	-	+	+	+	+
D-Fructose-6-PO_4	+	-	+	-	-	+
Tween 40	+	-	NA	-	+	+
β-Hydroxy-D,L-butyric acid	+	-	+	-	-	+
α-Keto-glutaric acid	+	+	-	+	+	+
Formic acid	+	+	NA	-	-	-
Dextrin	w	-	+	-	+	+
D,L-Lactic acid	w	+	+	-	+	+
Acetic acid	w	-	NA	-	-	+
Bromo-succinic acid	w	+	-	+	+	+
D-Sorbitol	-	-	NA	NA	+	+
D-Mannose	-	-	+	+	-	+
Maltose	-	-	+	+	+	+
N-Acetylglucosamine	-	-	-	-	+	-
L-Glutamic acid	-	-	+	+	+	+
Mannitol	-	-	+	+	+	+
Propionic acid	-	-	NA	-	+	-
L-Rhamnose	-	-	NA	+	-	+
D-Sucrose	-	-	NA	-	+	+
D-Cellobiose	-	-	+	NA	+	+
Gentiobiose	-	-	+	NA	NA	-

Supplementary table S4: Comparison of phenotypic characteristics of strain J223^{T} and its closest type strains of the family *Sphaerotilaceae*, and *Comamonadaceae*. Strains: 1) J223^{T}; 2) *Aquabacterium terrae* S2^{T} (11); 3) *Aquabacterium pictum* W35^{T} (12); 4) *Aquabacterium tepidiphilum* YIM 73027^{T} (13); 5) *Piscinibacter aquaticus* IMCC1728^{T} (14); 6) *Caldimonas thermodepolymerans* K14^{T} (15). Data for the reference strains were obtained from the available literature. All strains were Gram-stain negative. All species in this comparison (except C. *thermodepolymerans* where data are not available) were negative for β-glucuronidase, α-glucosidase, β-glucosidase, N-acetyl-β-glucosaminidase, α-mannosidase, α-fucosidase; and negative for utilization of D-Maltose, D-Sorbitol and D-Mannitol. +, Positive; −, negative; w, weak activity/utilization; NA, not analyzed or not determined.

	**1**	**2**	**3**	**4**	**5**	**6**
Isolation source	mineral water	soil	river biofilm	hot spring sediment	eutrophic pond	activated sludge
Cell size (µm)	0.5-0.6 x 0.9-2.1	0.7-1.0 x 1.6-3.2	0.7-1.0 × 1.6-2.7	1.0-1.3 x 1.6-2	0.8– 1.9 x 0.4–0.7	0.5-0.6 x 1.0-2.8
Motility	+	+	-	+	-	+
Flagella	single	single	none	single-polar	none	single-polar
Colony color	transparent-white	yellow	pink-beige	transparent	beige-milkish	white
**DNA G+C content (mol%)**	71.7	69.4	71.4	70.7	66.2	70.0
Growth range (optimum):						
Temperature	28-37 (32-37)	10-37 (28)	10-35 (30)	25-50 (37-45)	15-42 (30)	37-60 (45-50)
NaCl (%w/v)	≤4 (0.5-2)	0-3	≤0.5 (0)	0.5-1 (0.5)	≤1 (0)	NA
pH	6-9 (8-9)	5-9 (6.5-7)	7-9 (7-8)	6-8.5 (7-8)	6-10 (7)	6-9 (7)
**Basic enzymatic profile:**						
Oxidase	+	-	+	+	+	+
Catalase	-	-	+	+	-	+
Alkaline phosphatase	w	+	+	+	+	NA
Esterase (C4)	+	-	+	+	-	NA
Esterase-lipase (C8)	+	-	+	+	-	NA
Lipase (C14)	-	-	-	-	-	NA
Leucine-arylamidase	+	+	+	-	+	NA

	1	2	3	4	5	6
Valine-arylamidase	-	+	w	-	-	NA
Cystine-arylamidase	-	-	+	+	-	NA
Trypsin	-	+	+	-	-	NA
α-chymotrypsin	-	+	+	-	-	NA
Acid phosphatase	-	+	w	-	-	NA
Naphthol-AS-BI-phosphohydrolase	+	+	w	+	-	NA
α-galactosidase	-	+	-	-	-	NA
β-galactosidase	-	+	+	-	-	NA
**Assimilation of:**						
Dextrin	-	NA	+	-	NA	NA
D-Trehalose	-	NA	-	-	-	w
Sucrose	-	-	+	-	-	w
D-Turanose	-	NA	+	-	NA	NA
Stachyose	-	NA	-	-	NA	NA
N-Acetyl-D-glucosamine	-	-	-	-	+	NA
α-D-Glucose	-	-	-	-	-	+
D-Mannose	-	-	+	-	-	+
D-Fructose	-	NA	+	-	-	w
D-Galactose	-	NA	+	-	+	w
D-Fucose	-	NA	-	-	NA	NA
L-Rhamnose	-	-	-	-	NA	NA
D-Arabitol	-	NA	-	-	-	NA
Glycerol	-	NA	+	-	NA	NA
D-Glucose-6-PO_4	-	NA	+	-	NA	NA
D-Fructose-6-PO_4	+	NA	-	-	NA	NA

	1	2	3	4	5	6
D-Aspartic Acid	-	NA	-	-	NA	NA
D-Serine	-	NA	-	+	NA	NA
Gelatin	-	NA	-	-	NA	NA
Glycyl-L-Proline	-	-	-	+	NA	NA
Alanine	-	-	-	-	+	NA
L-Arginine	-	NA	-	+	+	NA
L-Aspartic Acid	-	NA	+	+	NA	NA
L-Glutamic Acid	-	NA	+	-	-	NA
L-Histidine	-	-	+	-	+	NA
L-Pyroglutamic Acid	-	NA	-	+	NA	NA
L-Serine	-	-	-	+	+	NA
Pectin	-	NA	-	-	NA	NA
Quinic Acid	-	NA	+	+	NA	NA
D-Saccharic Acid	-	NA	-	-	NA	NA
Methyl Pyruvate	-	NA	+	-	NA	NA
D-Lactic Acid Methyl Ester	-	NA	-	+	NA	NA
L-Lactic Acid	-	NA	+	-	NA	NA
α-Keto-glutaric Acid	+	NA	-	-	NA	NA
D-Malic Acid	-	-	-	-	+	NA
L-Malic Acid	-	NA	-	+	NA	NA
Tween 40	-	NA	+	-	NA	NA
α-Hydroxybutyric Acid	-	NA	-	+	NA	NA
β-Hydroxy- D,L-butyric Acid	-	NA	+	-	NA	NA
Acetoacetic Acid	+	NA	-	-	NA	NA

	1	2	3	4	5	6
Propionic Acid	-	-	-	+	+	NA
Acetic Acid	-	NA	+	+	NA	NA
Formic Acid	-	NA	+	+	NA	NA

Supplementary table S5: Comparison of phenotypic characteristics of strain J276^T and its closest type strains of the family *Burkholderiaceae* and *Comamonadaceae*. Strains: 1) J276^T; 2) *Aquincola rivuli* KYPY4^T (16); 3) *Aquincola tertiaricarbonis* L10^T (16, 17); 4) *Aquincola amnicola* TTM-94^T (18); 5) *Aquincola agrisoli* MAHUQ-54^T (19); 6) *Caldimonas thermodepolymerans* K14^T (15); 7) *Calidifontimicrobium sediminis* SYSU G00088^T (20). Data for the reference strains were obtained from the available literature. All strains are Gram-stain negative and motile by means of a polar flagellum. All strains (except C*. thermodepolymerans* where data are not available) were positive for oxidase, esterase (C4), esterase-lipase (C8), leucine-arylamidase and naphthol-AS-BI-phosphohydrolase, but negative for β-galactosidase. +, Positive; −, negative; w, weak activity/utilization; NA, not analyzed or not determined.

	1	2	3	4	5	6	7
Isolation source	mineral water	freshwater stream	contaminated groundwater	river water	rhizospheric soil	activated sludge	hot spring sediment
Cell size (µm)	0.5 x 1.5	0.6–0.7 x 0.9–1.3	0.8–1.1 x 1.2–2.3	0.6-1.0 x 1.0-1.1	0.5-1.1 x 1.2-2.7	0.5-0.6 x 1.0-2.8	0.6-0.7 x 1.0-1.6
Colony color	white	light-orange	white	cream	light pink	white	deep-yellow
DNA G+C content (mol%)	70.1	70.1	70.5	70.7	70.4	70.0	71.8
**Growth range (optimum):**							
Temperature (°C)	20-37 (24-28)	25-40 (30)	4-40 (30)	20-30 (30)	15-40 (30-35)	37-60 (45-50)	37-50
NaCl ($\%_{w/v}$)	≤1 (0.5)	≤0.5 (0)	≤1 (0)	≤2 (0.5)	0-0.5 (0)	NA	0.5
pH	7-10 (8-9)	6.5-7.5 (7)	5.5-8 (6-7)	6-8 (6)	5.5-9 (7)	6-9 (7)	5-9
**Basic enzymatic profile:**							
Catalase	+	+	+	w	+	+	+
Alkaline phosphatase	w	+	+	+	+	NA	+
Lipase (C14)	-	-	+	-	w	NA	-
Valine-arylamidase	+	-	+	-	+	NA	+
Cystine-arylamidase	-	-	+	+	+	NA	+
Trypsin	-	+	-	-	w	NA	w
α-chymotrypsin	-	+	-	-	+	NA	-
Acid phosphatase	w	+	+	+	+	NA	-
α-galactosidase	-	-	-	+	-	NA	-
β-glucuronidase	-	-	+	-	-	NA	NA
α-glucosidase	+	-	+	+	-	NA	-

	1	2	3	4	4	5	6
β-glucosidase	-	-	+	-	-	NA	-
N-acetyl-β-glucosaminidase	-	-	-	-	w	NA	NA
α-mannosidase	-	-	-	-	-	NA	NA
α-fucosidase	-	-	-	-	-	NA	NA
**<u>Assimilation of:</u>**							
Dextrin	+	+	-	-	NA	NA	NA
D-Maltose	+	+	+	-	-	NA	+
D-Trehalose	+	+	-	-	NA	w	NA
Sucrose	+	-	+	-	NA	w	NA
D-Turanose	+	-	+	-	NA	NA	+
N-Acetyl-D-glucosamine	+	-	+	-	-	NA	-
α-D-Glucose	+	-	+	-	-	+	-
D-Mannose	+	-	+	-	-	+	-
D-Fructose	+	-	+	-	NA	w	+
D-Sorbitol	+	-	+	-	NA	NA	NA
D-Mannitol	+	-	+	-	+	NA	-
D-Arabitol	+	-	+	-	NA	NA	NA
Glycerol	+	-	+	-	NA	NA	NA
L-Alanine	-	+	+	-	NA	NA	NA
L-Aspartic Acid	+	-	+	+	NA	NA	NA
L-Glutamic Acid	+	+	-	+	NA	NA	NA
L-Histidine	-	-	+	-	NA	NA	NA
L-Pyroglutamic Acid	+	+	-	-	NA	NA	NA
L-Serine	-	+	+	-	NA	NA	NA
D-Gluconic Acid	+	+	+	-	NA	NA	NA
D-Glucuronic Acid	-	+	+	-	NA	NA	+
Glucuronamide	-	+	-	+	NA	NA	+

	1	2	3	4	4	5	6
Quinic Acid	+	-	+	-	NA	NA	NA
D-Saccharic Acid	+	+	-	-	NA	NA	NA
α-Keto-glutaric Acid	-	+	+	NA	NA	NA	+
D-Malic Acid	+	-	-	+	NA	NA	+
Bromo-succinic Acid	-	-	+	+	NA	NA	NA
γ-Amino-butyric Acid	+	-	+	-	NA	NA	NA
Propionic Acid	-	-	+	+	NA	NA	NA
Acetic Acid	+	-	-	+	NA	NA	NA
Formic Acid	-	+	-	+	NA	NA	NA

Supplementary table S6: Comparison of phenotypic characteristics of strain J367^T, J426^T and their closest type strains of the family *Caulobacteraceae*. Stains: 1) J367^T; 2) J426^T; 3) Phenylobacterium immobile E^T (21-23); 4) *Phenylobacterium muchangponense* A8^T (22); 5) *Phenylobacterium kunshanense* BUT-10^T (24); 6) *Phenylobacterium panacis* DCY109^T (23). Data for the reference strains were obtained from the available literature. All strains are Gram-stain negative. All strains were positive for catalase, alkaline phosphatase but negative for lipase (C14), α-galactosidase, β-glucuronidase, α-fucosidase and assimilation of D-maltose, sucrose, α-D-glucose, D-mannose, D-sorbitol, D-mannitol, L-alanine, L-histidine, D-malic acid. +, Positive; −, negative; w, weak activity/utilization; NA, not analyzed or not determined.

	**1**	**2**	**3**	**4**	**5**	**6**
Isolation source	mineral water	mineral water	soil	soil	activated sludge	rhizosphere
Cell size (µm)	0.5 x 1.8	0.5 x 0.8	0.7-1.0 x 1-2	0.5-0.6 x 0.7-1.3	0.4-0.45 x 0.9-1.4	0.4-0.5 x 0.4-1.3
Motility	-	-	-	-	+	-
Flagella	none	none	none	none	single polar	none
Colony color	white	white	greenish yellow	colorless	white	white
DNA G+C content (mol%)	69.96	69.04	65.5	72.3	71.85	66.9
**Growth range (optimum):**						
Temperature (°C)	20-37 (30)	12-37 (30)	28-30	15-40 (25-35)	25-37 (28-30)	20-30 (25)
NaCl (%w/v)	≤1.5 (0.5)	≤2.5 (1)	≤1	≤0.5 (0)	≤0.5	0
pH	6-10 (8-9)	6-10 (7-8)	6.5-8 (6.8-7)	5.5-9.5 (7)	6-8 (7)	6-9 (7)
**Basic enzymatic profile:**						
Oxidase	-	-	w	+	+	-
Esterase (C4)	+	+	+	w	+	+
Esterase-lipase (C8)	w	+	+	w	+	+
Leucine-arylamidase	+	+	+	w	+	+
Valine-arylamidase	+	+	w	w	+	+
Cystine-arylamidase	-	+	-	w	-	+
Trypsin	+	+	-	w	+	-
α-chymotrypsin	+	+	-	-	-	+
Acid phosphatase	+	+	+	-	+	+

	1	2	3	4	5	6
Naphthol-AS-BI-phosphohydrolase	+	+	+	-	+	+
β-galactosidase	+	-	-	-	-	-
α-glucosidase	+	+	w	-	+	-
β-glucosidase	-	-	-	-	+	+
N-acetyl-β-glucosaminidase	+	-	-	+	+	+
α-mannosidase	w	-	-	-	-	+
**Assimilation of:**						
D-Trehalose	-	-	NA	NA	-	NA
D-Cellobiose	-	-	NA	NA	-	NA
Gentiobiose	-	-	NA	NA	-	NA
N-Acetyl-D-glucosamine	-	-	-	w	-	-
D-Fructose	-	-	NA	NA	-	NA
D-Galactose	-	-	NA	+	-	NA
D-Fucose	-	-	NA	NA	-	NA
L-Rhamnose	-	-	-	-	-	+
D-Arabitol	-	-	NA	NA	-	NA
D-Serine	-	-	NA	NA	+	NA
L-Serine	-	-	-	-	+	+
Propionic Acid	-	-	-	w	+	-
Acetic Acid	+	-	-	-	NA	NA

Supplementary table S7: Comparison of phenotypic characteristics of strain J372^T and its closest type strains of the family *Flavobacteriaceae*. Stains: 1) J372^T; 2) *Flavobacterium album* HYN0059^T (25); 3) *Flavobacterium hauense* BX12^T (26); 4) *Flavobacterium akiainvivens* IK-1^T (27); 5) *Flavobacterium subsaxonicum* WB 4.1-42^T (28); 6) *Flavobacterium cyanobacteriorum* TH021^T (29). Data for the reference strains were obtained from the available literature. All strains are Gram-stain negative and non-motile. All strains were positive for leucine-arylamidase, but negative for α-chymotrypsin, N-acetyl-β-glucosaminidase and utilization of N-Acetyl-D-Glucosamine. +, Positive; −, negative; w, weak activity/utilization; NA, not analyzed or not determined.

	**1**	**2**	**3**	**4**	**5**	**6**
Isolation source	mineral water	freshwater	soil	decaying wood	freshwater	cyanobacterial aggregates
Cell size (µm)	0.6 x 2	0.4-0.5 x 1-1.45	0.4-0.6 x 0.9-3.6	0.4 x 2	1.5 x 3.5-4.5	0.6-1.0 x 1-1.8
Colony color	yellow	white	yellow	cream-white	light yellow	yellow
DNA G+C content (mol%)	42.4	44.5	43.9	44.2	43.3	41.9
**Growth range (optimum):**						
Temperature (°C)	20-32 (30)	4-40 (30)	4-32 (28)	30-35	2-38 (24-28)	4-32 (28)
NaCl ($\%_{w/v}$)	≤0.5	≤2 (0)	≤2 (0)	≤2	≤2 (0)	≤1 (0)
pH	6-11	5.5.-7.5 (7)	6-8 (7)	NA	7-7.6 (7.2)	5-10 (7)
**Basic enzymatic profile:**						
Oxidase	-	+	+	-	+	+
Catalase	-	-	-	+	+	+
Alkaline phosphatase	+	w	+	+	+	+
Esterase (C4)	+	-	+	+	-	+
Esterase-lipase (C8)	+	-	+	+	w	+
Lipase (C14)	-	-	-	+	-	-
Valine-arylamidase	+	-	+	+	+	+
Cystine-arylamidase	+	-	-	+	w	+
Trypsin	+	-	-	w	+	+
Acid phosphatase	-	w	+	w	+	+
Naphthol-AS-BI-phosphohydrolase	+	-	+	+	+	+
α-galactosidase	-	-	+	+	+	-
β-galactosidase	-	-	-	+	+	-
β-glucuronidase	+	-	-	NA	-	-

	1	2	3	4	5	6
α-glucosidase	+	+	-	+	+	+
β-glucosidase	w	-	-	+	-	-
α-mannosidase	-	-	-	NA	-	-
α-fucosidase	-	-	-	NA	-	-
**Assimilation of:**						
D-Maltose	+	-	+	w	+	-
D-Trehalose	-	NA	NA	+	-	NA
D-Cellobiose	-	NA	NA	+	+	NA
Gentiobiose	+	NA	NA	+	+	NA
Sucrose	-	NA	-	w	-	-
D-Turanose	-	NA	NA	w	w	NA
α-D-Glucose	+	-	+	+	+	-
D-Mannose	+	-	+	+	+	-
D-Fructose	-	NA	NA	+	+	NA
D-Galactose	-	NA	NA	w	+	NA
L-Rhamnose	-	NA	-	w	+	NA
D-Sorbitol	-	NA	-	NA	-	NA
D-Mannitol	-	NA	-	NA	-	-
L-Alanine	-	NA	-	+	+	NA
L-Arginine	-	NA	NA	+	-	NA
L-Histidine	-	NA	-	NA	-	NA
L-Serine	-	NA	-	+	+	NA
D-Galacturonic Acid	+	NA	NA	w	-	NA
D-Glucuronic Acid	-	NA	NA	w	-	NA
D-Malic Acid	-	NA	-	NA	-	-
α-Keto-butyric Acid	-	NA	NA	+	+	NA
Propionic Acid	-	NA	-	NA	-	NA
Acetic Acid	+	NA	NA	+	-	NA

Supplementary table S8: Comparison of phenotypic characteristics of strain J428[T] and its closest type strains of the family *Phyllobacteriaceae*. Stains: 1) 428[T]; 2) *Mesorhizobium qingshengii* CCBAU 33460[T] (30); 3) *Mesorhizobium shangrilense* CCBAU 65327[T] (31); 4) *Mesorhizobium cantuariense* CCBAU 19515[T] (32); 5) *Mesorhizobium newzealandse* ICMP 19545[T] (33); 6) *Mesorhizobium calcicola* ICMP 19560[T] (33). Data for the reference strains were obtained from the available literature. All strains are Gram-stain negative. All strains were positive for assimilation of α-D-Glucose and D-Mannose. +, Positive; −, negative; w, weak activity/utilization; NA, not analyzed or not determined.

	1	2	3	4	5	6
Isolation source	mineral water	root nodules	root nodules	root nodules	root nodules	root nodules
Cell size (μm)	0.5 x 1.0	0.38-0.52 x 60.75-2.12	0.3-0.6 x 1-3	0.5-0.8 x 1–2	0.6-0.7 x 1.5-2.5	1 x 2
Motility	+	NA	NA	NA	NA	NA
Flagella	multiple monopolar	NA	NA	NA	NA	NA
Colony color	white	NA	white	white	white	white
DNA G+C content (mol%)	65.1	59.5	61.4	62.5	62.6	62.6
**Growth range (optimum):**						
Temperature (°C)	20-32 (30)	10-35 (28)	10-37 (28)	15-30	15-30	15-30
NaCl ($\%_{w/v}$)	0.5-2 (1)	≤1	≤3	1-8	1-8	1-7
pH	6-11 (8-9)	5.5-9 (7)	5-10 (7)	4-9	4-9	6-9
**Basic enzymatic profile:**						
Oxidase	-	NA	+	-	+	+
Catalase	-	+	+	+	-	-
β-galactosidase	-	NA	NA	+	NA	NA
β-glucosidase	-	NA	NA	+	NA	NA
**Assimilation of:**						
Dextrin	-	-	-	w	w	w
D-Maltose	-	+	+	w	+	+
D-Trehalose	-	NA	+	+	+	+
D-Cellobiose	+	NA	NA	+	+	+
Gentiobiose	+	NA	NA	+	+	+
Sucrose	-	+	+	+	w	+
D-Turanose	-	NA	-	+	w	+

	1	2	3	4	5	6
Stachyose	-	NA	NA	-	-	-
N-Acetyl-D-glucosamine	-	NA	NA	+	+	+
D-Fructose	+	+	+	w	+	+
D-Galactose	+	+	-	+	+	+
D-Fucose	+	NA	NA	+	+	+
L-Rhamnose	-	-	+	+	+	+
D-Sorbitol	+	-	+	+	-	+
D-Mannitol	+	NA	NA	+	w	+
D-Arabitol	+	NA	NA	w	+	+
Glycerol	+	NA	NA	+	+	+
D-Glucose-6-PO_4	-	NA	NA	w	-	-
D-Fructose-6-PO_4	-	NA	NA	w	-	-
D-Aspartic Acid	-	NA	NA	w	w	-
D-Serine	-	NA	NA	-	-	-
Gelatin	-	NA	NA	-	-	-
Glycyl-L-proline	-	NA	NA	w	w	w
L-Alanine	-	NA	NA	w	+	+
L-Arginine	-	+	-	+	+	+
L-Aspartic Acid	-	NA	NA	+	+	-
L-Glutamic Acid	-	NA	NA	+	w	+
L-Histidine	-	NA	NA	+	w	-
L-Pyroglutamic Acid	-	NA	NA	w	-	-
L-Serine	-	NA	NA	-	-	-
Pectin	-	NA	NA	w	-	w
D-Galacturonic Acid	+	NA	NA	w	w	-
L-Galactonic Acid Lactone	-	NA	NA	-	-	-
D-Gluconic Acid	-	-	NA	w	-	-
D-Glucuronic Acid	+	NA	NA	w	w	-

	1	2	3	4	5	6
Glucuronamide	-	NA	NA	w	w	w
Quinic Acid	+	NA	NA	w	-	-
D-Saccharic Acid	-	NA	NA	+	-	-
Methyl Pyruvate	-	NA	NA	w	-	-
D-Lactic Acid Methyl Ester	+	NA	NA	w	-	-
L-Lactic Acid	+	NA	NA	+	+	+
α-Keto-glutaric Acid	-	NA	NA	w	-	-
D-Malic Acid	-	-	NA	+	-	+
L-Malic Acid	-	NA	NA	+	+	+
Bromo-succinic Acid	-	NA	NA	+	-	-
Tween 40	+	NA	NA	w	w	-
γ-Amino-butyric Acid	-	NA	NA	+	+	+
α-Hydroxybutyric Acid	-	NA	NA	w	-	-
β-Hydroxy-D,L-butyric Acid	-	NA	NA	+	+	w
α-Keto-butyric Acid	-	NA	NA	w	-	-
Acetoacetic Acid	+	NA	NA	+	+	+
Propionic Acid	-	NA	NA	+	+	w
Acetic Acid	-	NA	NA	+	+	+
Formic Acid	-	-	NA	-	w	-

Supplementary table S9: Comparison of phenotypic characteristics of strain J452^T and its closest type strains of the family *Pseudomonadaceae*. Stains: 1) J452^T; 2) *Pseudomonas campi* S1-A32-2^T (34); 3) *Pseudomonas guryensis* SR9^T (35); 4) *Pseudomonas ullengensis* UL070^T (35); 5) *Pseudomonas peli* LMG 23201^T (36); 6) *Pseudomonas leptonychotis* P5773^T (37). Data for the reference strains were obtained from the available literature. All strains are Gram-stain negative. All strains were positive for esterase-lipase (C8) and leucine-arylamidase, but negative for α-chymotrypsin, α-galactosidase, β-galactosidase, β-glucuronidase, α-glucosidase, β-glucosidase, N-acetyl-β-glucosaminidase, α-mannosidase and α-fucosidase. +, Positive; −, negative; w, weak activity/utilization; NA, not analyzed or not determined.

	1	2	3	4	5	6
Isolation source	mineral water	soil	riverside soil	island soil	nitrifying inoculum	animal
Cell size (μm)	0.4 x 1.2	NA	0.4-0.8 x 1.5-2	0.4-0.8 x 1.5-2	1 x 2-3	0.5-0.7 x 2-4
Motility	+	+	NA	+	+	+
Flagella	single	NA	NA	single	NA	single/multiflagellar, polar
Colony color	white	transparent	NA	NA	beige-yellow	yellowish
DNA G+C content (mol%)	63.2	63.5	63.2	63.6	60.7	58.8
**Growth range (optimum):**						
Temperature (°C)	20-37 (32)	25-37 (25)	10-37 (30)	4-33 (30)	28-37	2-35 (25)
NaCl ($\%_{w/v}$)	0.5-2 (1)	≤2	≤1 (0)	≤2 (0)	NA	0.5-3 (1)
pH	6-11	6-9	4-10 (8)	4-10 (7)	NA	6-8 (7)
**Basic enzymatic profile:**						
Oxidase	+	+	NA	NA	+	+
Catalase	-	+	NA	NA	+	+
Alkaline phosphatase	w	-	-	+	-	+
Esterase (C4)	+	+	+	+	-	+
Lipase (C14)	+	+	+	+	w	+
Valine-arylamidase	-	w	w	w	-	w
Cystine-arylamidase	-	NA	-	-	-	-
Trypsin	-	-	+	+	-	-
Acid phosphatase	+	-	+	+	-	+
Naphthol-AS-BI-phosphohydrolase	w	+	+	+	+	-

	1	2	3	4	5	6
**Assimilation of:**						
D-Maltose	-	-	NA	NA	-	-
D-Trehalose	-	-	NA	NA	-	-
D-Cellobiose	-	-	NA	NA	NA	-
Gentiobiose	-	-	NA	NA	-	w
Sucrose	-	-	NA	NA	-	-
D-Turanose	-	-	NA	NA	-	-
Stachyose	-	-	NA	NA	NA	-
N-Acetyl-D-glucosamine	-	-	NA	NA	-	-
α-D-Glucose	-	-	NA	NA	-	-
D-Mannose	-	-	NA	NA	-	-
D-Fructose	-	-	NA	NA	-	w
D-Galactose	-	-	NA	NA	-	-
L-Rhamnose	-	-	NA	NA	NA	w
D-Sorbitol	-	-	NA	NA	NA	-
D-Mannitol	-	-	NA	NA	-	-
D-Arabitol	-	-	NA	NA	-	-
Glycerol	-	-	NA	NA	-	-
D-Glucose-6-PO_4	-	-	NA	NA	NA	-
D-Fructose-6-PO_4	-	-	NA	NA	NA	+
D-Aspartic Acid	-	-	NA	NA	NA	-
D-Serine	-	-	NA	NA	NA	+
Gelatin	-	w	NA	NA	NA	-
Glycyl-L-proline	-	-	NA	NA	NA	-
L-Alanine	+	+	NA	NA	NA	+
L-Arginine	+	+	NA	NA	+	+
L-Aspartic Acid	+	+	NA	NA	NA	+
L-Glutamic Acid	+	+	NA	NA	NA	+

	1	2	3	4	5	6
L-Histidine	+	+	NA	NA	NA	+
L-Pyroglutamic Acid	-	-	NA	NA	NA	-
L-Serine	+	-	NA	NA	NA	-
Pectin	-	-	NA	NA	NA	w
D-Galacturonic Acid	-	-	NA	NA	NA	w
L-Galactonic Acid Lactone	-	-	NA	NA	NA	w
D-Gluconic Acid	-	-	NA	NA	NA	-
Glucuronamide	-	w	NA	NA	NA	w
Quinic Acid	-	-	NA	NA	NA	-
D-Saccharic Acid	-	-	NA	NA	NA	-
Methyl Pyruvate	+	w	NA	NA	NA	+
D-Lactic Acid Methyl Ester	-	-	NA	NA	NA	-
L-Lactic Acid	-	-	NA	NA	NA	+
α-Keto-glutaric Acid	-	-	NA	NA	NA	w
D-Malic Acid	-	-	NA	NA	NA	+
L-Malic Acid	+	+	NA	NA	+	+
Bromo-succinic Acid	+	-	NA	NA	NA	+
Tween 40	+	+	NA	NA	NA	+
γ-Amino-butyric Acid	+	-	NA	NA	NA	+
α-Hydroxybutyric Acid	-	+	NA	NA	NA	w
β-Hydroxy-D,L-butyric Acid	+	w	NA	NA	NA	+
α-Keto-butyric Acid	-	-	NA	NA	NA	w
Acetoacetic Acid	-	w	NA	NA	NA	+
Propionic Acid	+	+	NA	NA	NA	+
Acetic Acid	+	+	NA	NA	NA	+
Formic Acid	+	+	NA	NA	NA	-

Supplementary table S10: Fatty acid profile of Jáchymov strains. Fatty acids representing ≤1.0% of the total fatty acids are not reported. The symbol — is used to indicate a concentration of zero.

	**J193^T**	**J219^T**	**J223^T**	**J276^T**	**J367^T**	**J372^T**	**J426^T**	**J428^T**	**J452^T**
$C_{14:0}$	—	1.3	—	—	—	—	—	—	—
iso-$C_{15:0}$	4.6	—	—	—	—	**47.9**	—	1.7	—
anteiso-$C_{15:0}$	—	—	—	—	—	**10.1**	—	—	—
$C_{15:0}$	—	—	—	7.6	1.2	6.0	3.6	—	2.4
iso-$C_{16:0}$	—	—	—	—	—	**13.6**	—	—	—
$C_{16:1}$ ω7c	**39.1**	**16.5**	3.4	**14.3**	1.6	3.6	—	1.5	**30.7**
$C_{16:1}$ ω5c	1.8	—	—	—	—	—	—	—	—
$C_{16:0}$	**52.8**	**25.4**	**37.4**	**29.4**	**20.8**	4.1	**13.5**	**13.9**	**16.6**
iso-$C_{17:0}$	—	—	—	—	—	—	—	4.9	—
cyclo-$C_{17:0}$	—	**32.6**	**17.3**	**31.7**	—	—	4.4	2.0	—
$C_{17:0}$	—	4.3	—	4.7	1.1	—	9.2	3.7	2.0
$C_{18:1}$ ω7c	—	**16.7**	—	9.4	**72.4**	—	**64.2**	**55.3**	**42.5**
$C_{18:0}$	—	1.3	**39.6**	1.4	—	1.7	—	**11.2**	1.1

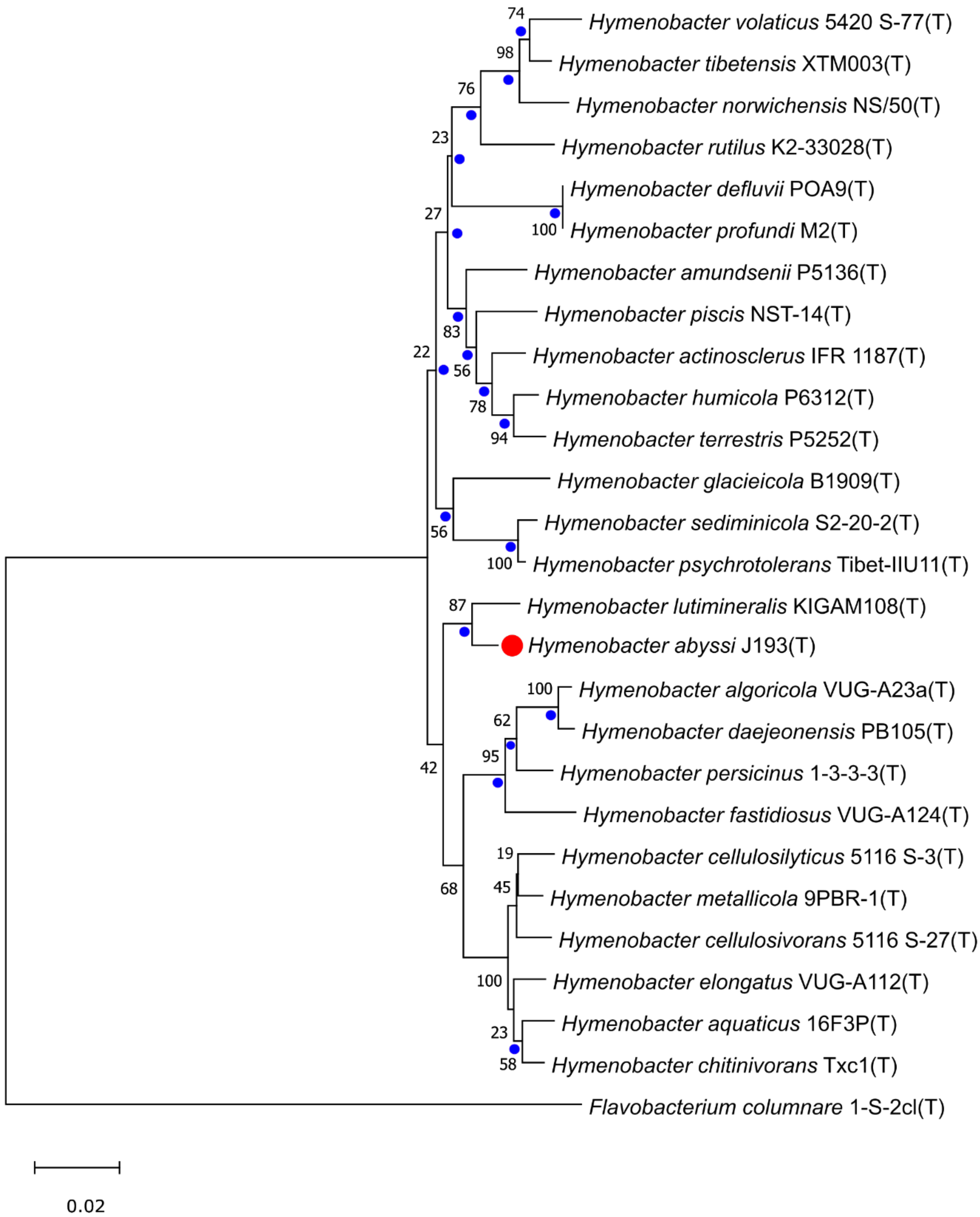

Figure S1: Phylogeny reconstruction of strain J193[T] and its closest type strains based on secondary structure of 16S rRNA gene. The evolutionary history was inferred using the Neighbor-Joining method (38) based on the Kimura 2-parameter model (39). Bootstrapping was used to test the topology of the tree (1000 replicates), the percentage of trees in which the associated taxa clustered together is indicated. A discrete Gamma distribution was used to model evolutionary rate differences among sites (5 categories). A total of 1,288 positions were used in the final dataset. The blue dots show where evolutionary branches converge, as determined using the Maximum Likelihood method (40) with 1,000 replicates. Evolutionary analyses were conducted in MEGA11 (41). The bar represents the number of substitutions per site.

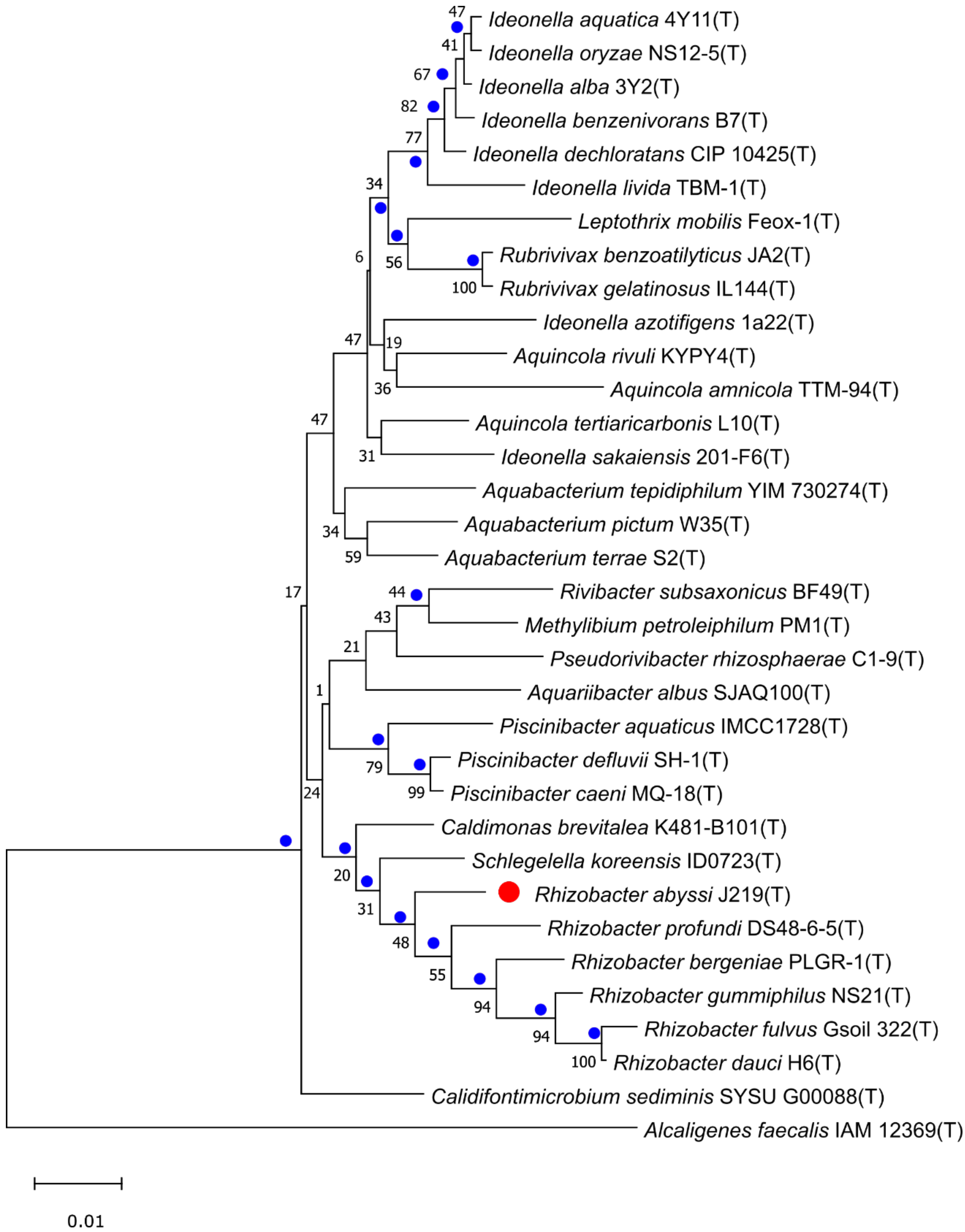

Figure S2: Phylogeny reconstruction of strain J219^T and its closest type strains based on secondary structure of 16S rRNA gene. The evolutionary history was inferred using the Neighbor-Joining method (38) based on the Kimura 2-parameter model (39). Bootstrapping was used to test the topology of the tree (1000 replicates), the percentage of trees in which the associated taxa clustered together is indicated. A discrete Gamma distribution was used to model evolutionary rate differences among sites (5 categories). A total of 1,329 positions were used in the final dataset. The blue dots show where evolutionary branches converge, as determined using the Maximum Likelihood method (40) with 1,000 replicates. Evolutionary analyses were conducted in MEGA11 (41). The bar represents the number of substitutions per site.

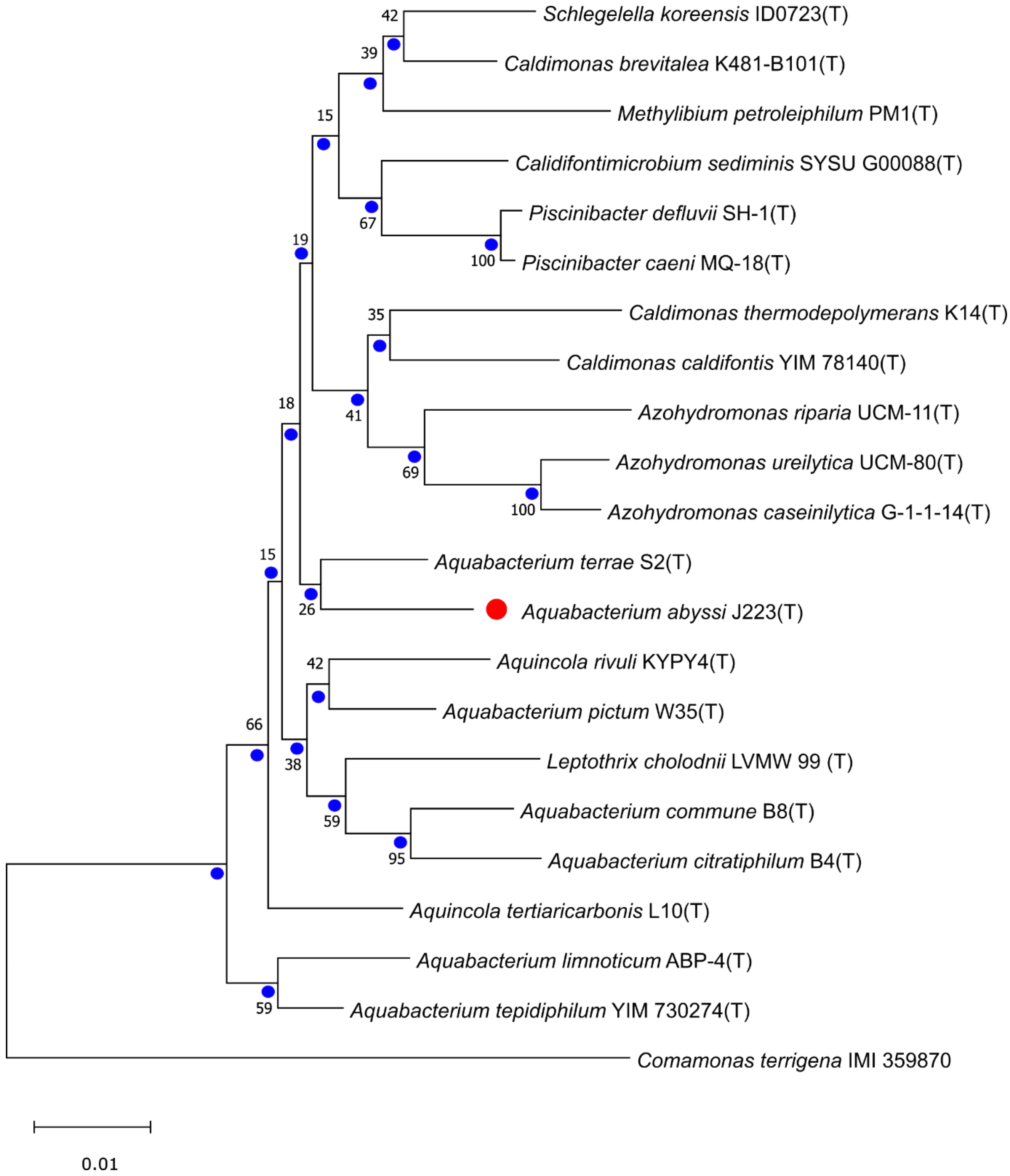

Figure S3: Phylogeny reconstruction of strain J223^{T} and its closest type strains based on secondary structure of 16S rRNA gene. The evolutionary history was inferred using the Neighbor-Joining method (38) based on the Kimura 2-parameter model (39). Bootstrapping was used to test the topology of the tree (1000 replicates), the percentage of trees in which the associated taxa clustered together is indicated. A discrete Gamma distribution was used to model evolutionary rate differences among sites (5 categories). A total of 1,371 positions were used in the final dataset. The blue dots show where evolutionary branches converge, as determined using the Maximum Likelihood method (40) with 1,000 replicates. Evolutionary analyses were conducted in MEGA11 (41). The bar represents the number of substitutions per site.

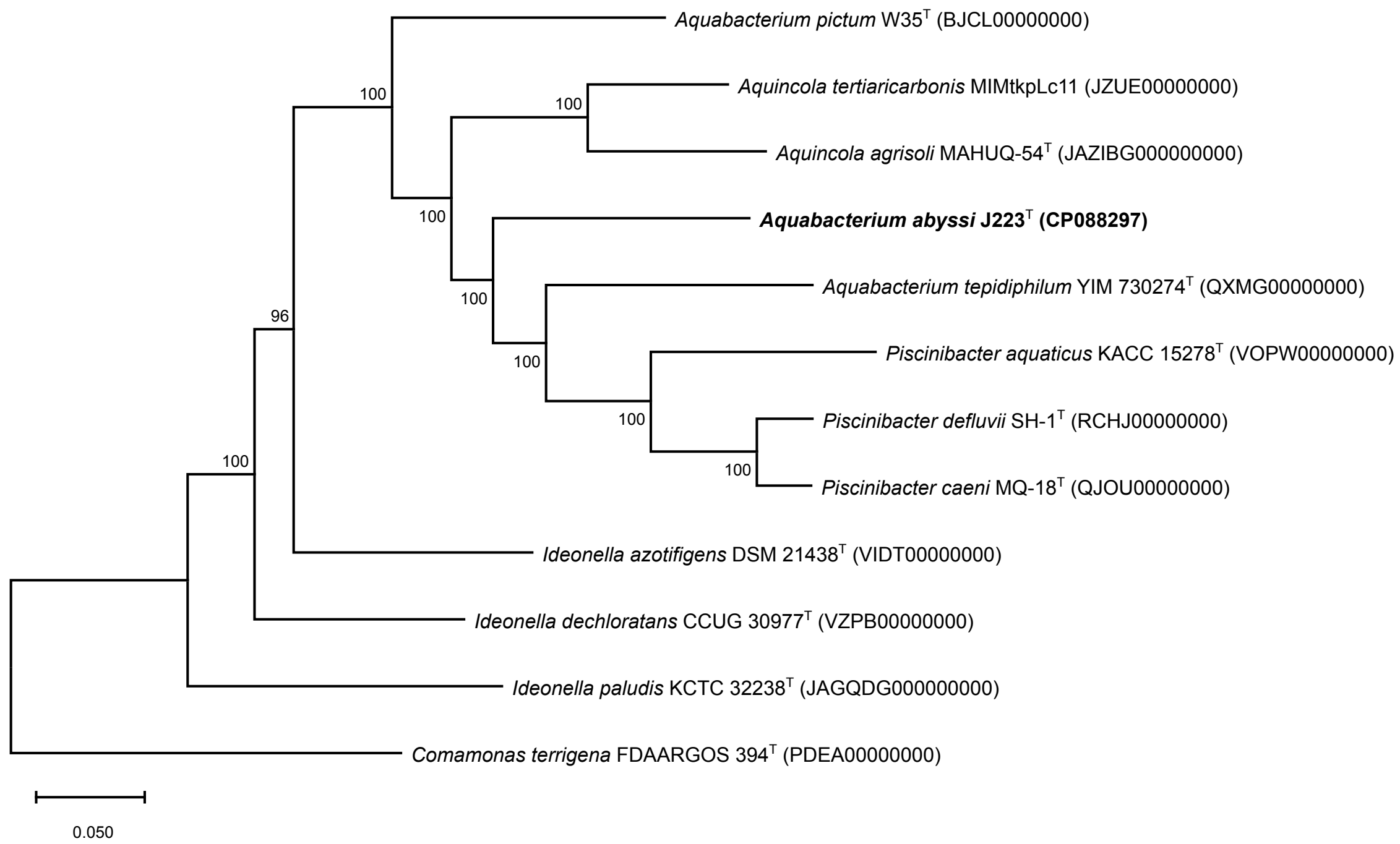

Figure S4: UBCG based core-genome phylogenetic analysis (targeting 92 housekeeping genes) of the strain J223^T with its closest members from the family *Comamonadaceae*.

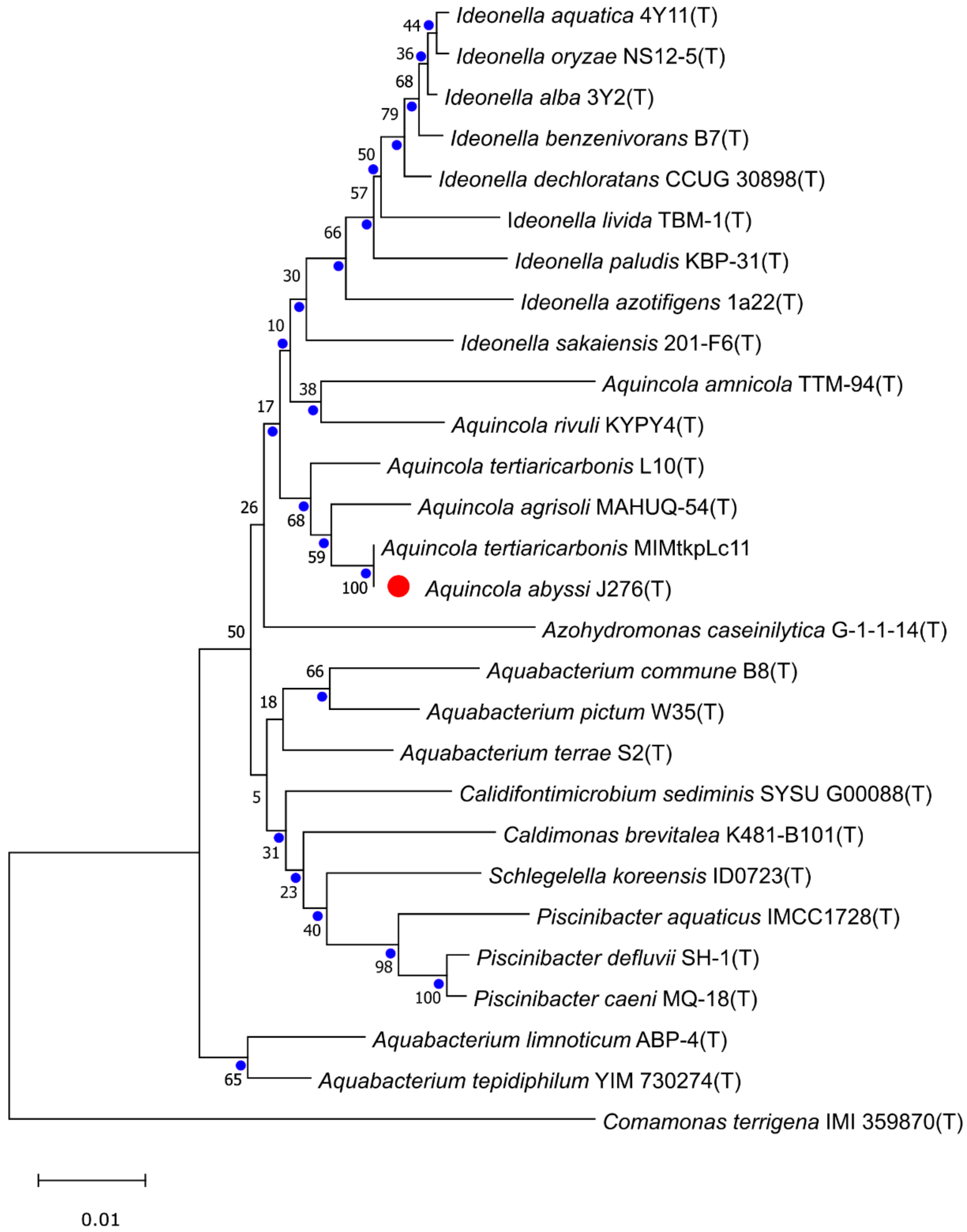

Figure S5: Phylogeny reconstruction of strain J276^T and its closest type strains based on secondary structure of 16S rRNA gene. The evolutionary history was inferred using the Neighbor-Joining method (38) based on the Kimura 2-parameter model (39). Bootstrapping was used to test the topology of the tree (1000 replicates), the percentage of trees in which the associated taxa clustered together is indicated. A discrete Gamma distribution was used to model evolutionary rate differences among sites (5 categories). A total of 1,330 positions were used in the final dataset. The blue dots show where evolutionary branches converge, as determined using the Maximum Likelihood method (40) with 1,000 replicates. Evolutionary analyses were conducted in MEGA11 (41). The bar represents the number of substitutions per site.

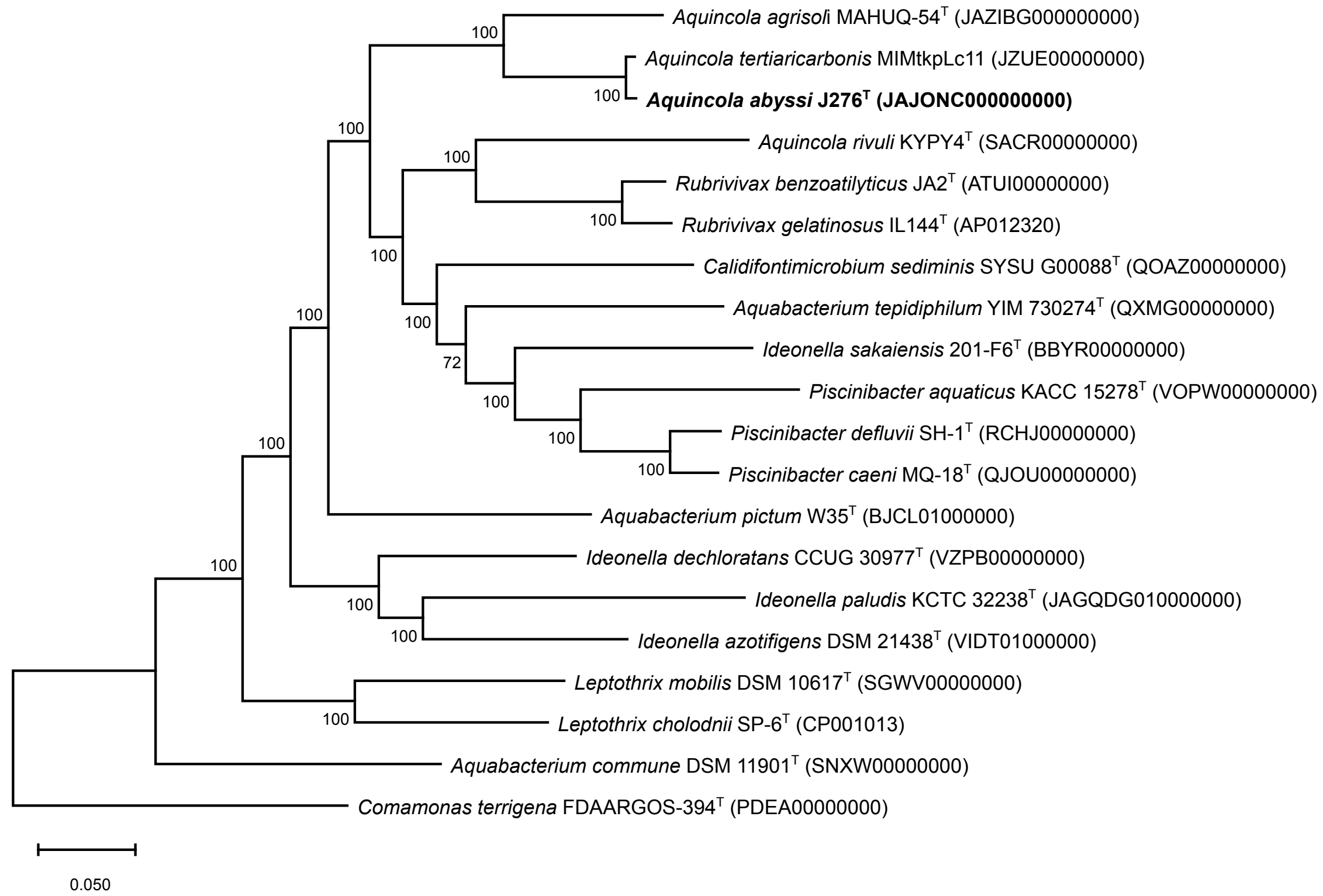

Figure S6: UBCG based core-genome phylogenetic analysis (targeting 92 housekeeping genes) of strain J276^T with its closest members from the family *Burkholderiaceae*.

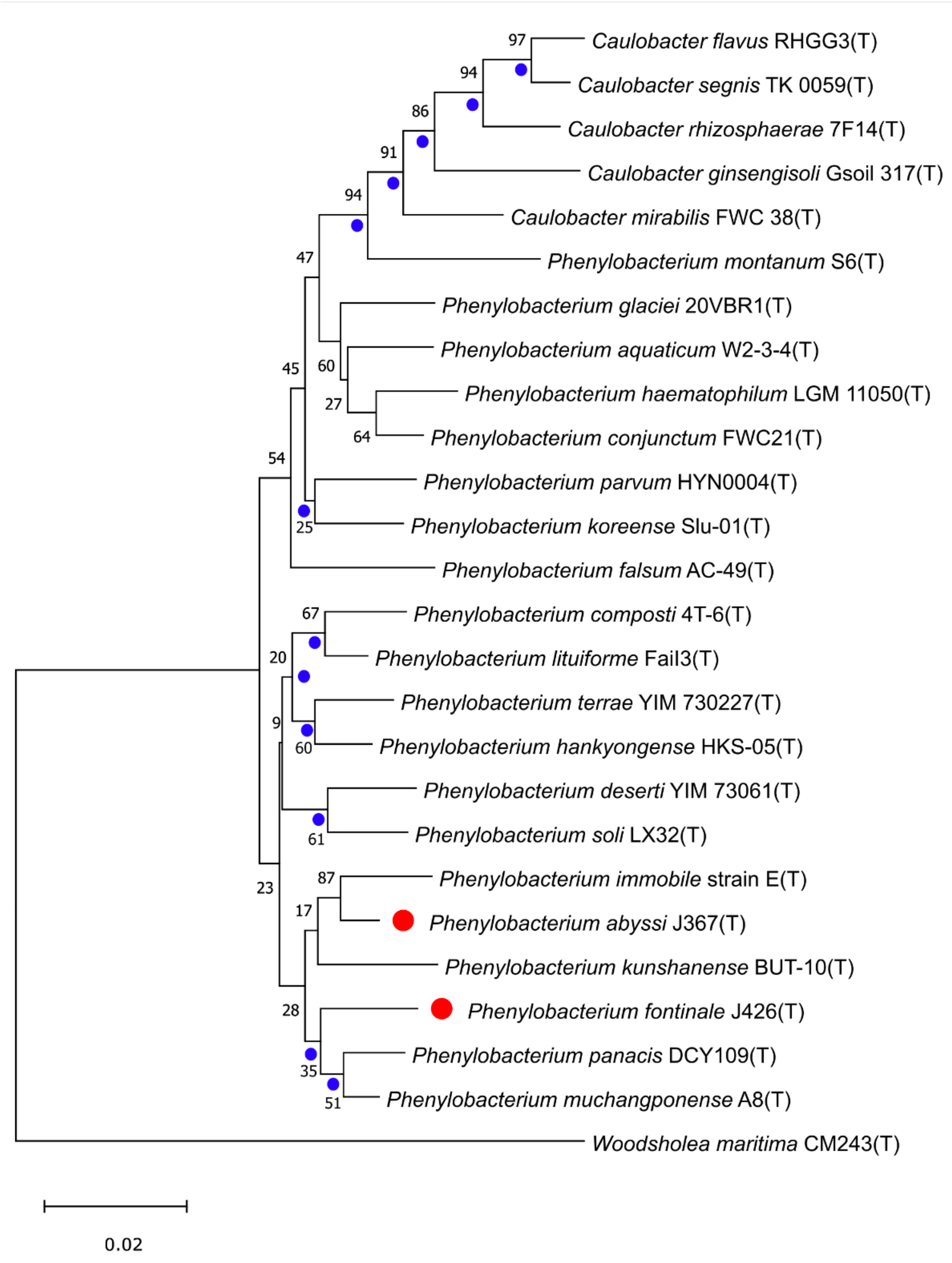

Figure S7: Phylogeny reconstruction of strain J376^{T}, J426^{T} and their closest type strains based on secondary structure of 16S rRNA gene. The evolutionary history was inferred using the Neighbor-Joining method (38) based on the Kimura 2-parameter model (39). Bootstrapping was used to test the topology of the tree (1000 replicates), the percentage of trees in which the associated taxa clustered together is indicated. A discrete Gamma distribution was used to model evolutionary rate differences among sites (5 categories). A total of 1,337 positions were used in the final dataset. The blue dots show where evolutionary branches converge, as determined using the Maximum Likelihood method (40) with 1,000 replicates. Evolutionary analyses were conducted in MEGA11 (41). The bar represents the number of substitutions per site.

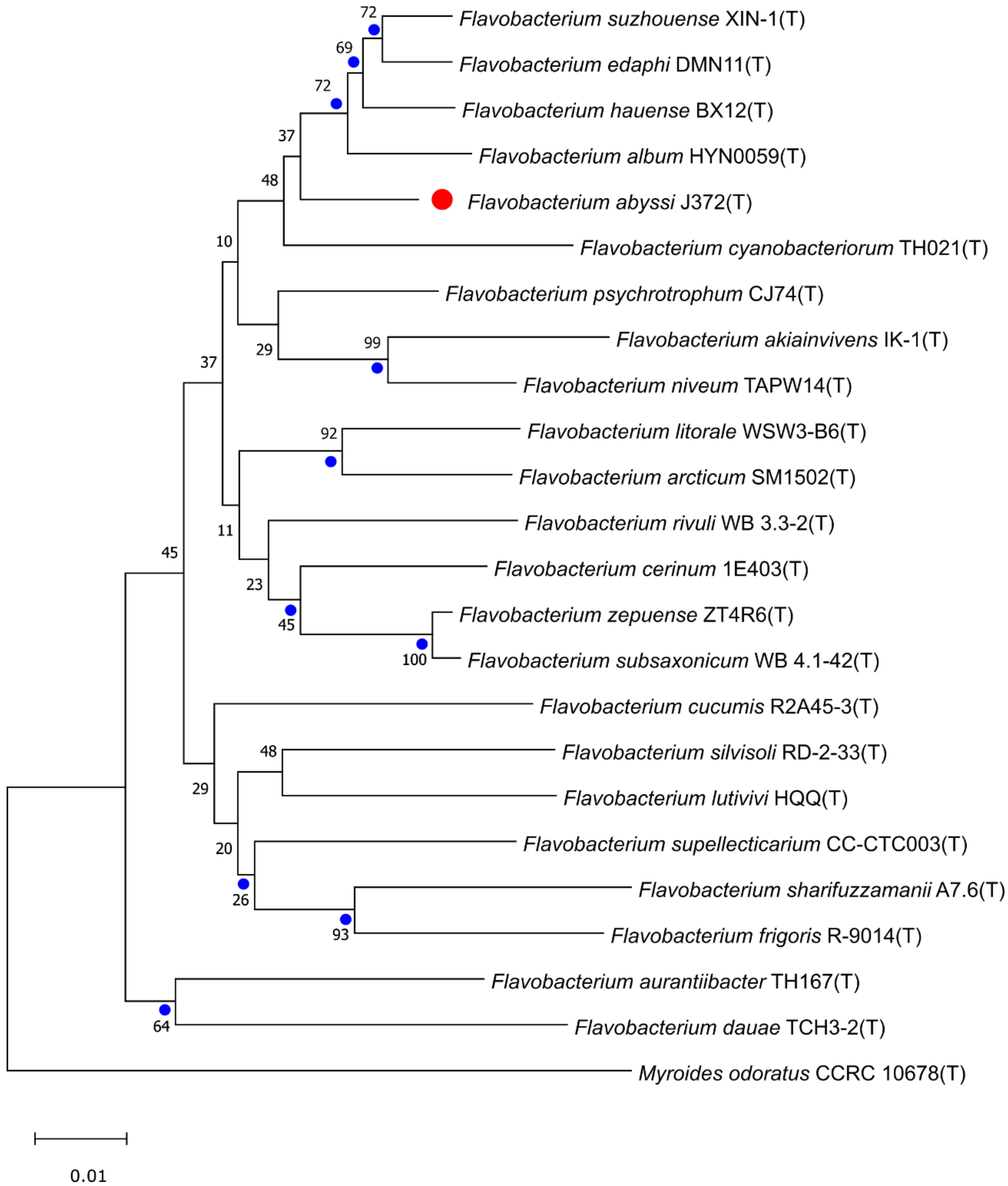

Figure S8: Phylogeny reconstruction of strain J372^T and its closest type strains based on secondary structure of 16S rRNA gene. The evolutionary history was inferred using the Neighbor-Joining method (38) based on the Kimura 2-parameter model (39). Bootstrapping was used to test the topology of the tree (1000 replicates), the percentage of trees in which the associated taxa clustered together is indicated. A discrete Gamma distribution was used to model evolutionary rate differences among sites (5 categories). A total of 1,341 positions were used in the final dataset. The blue dots show where evolutionary branches converge, as determined using the Maximum Likelihood method (40) with 1,000 replicates. Evolutionary analyses were conducted in MEGA11 (41). The bar represents the number of substitutions per site.

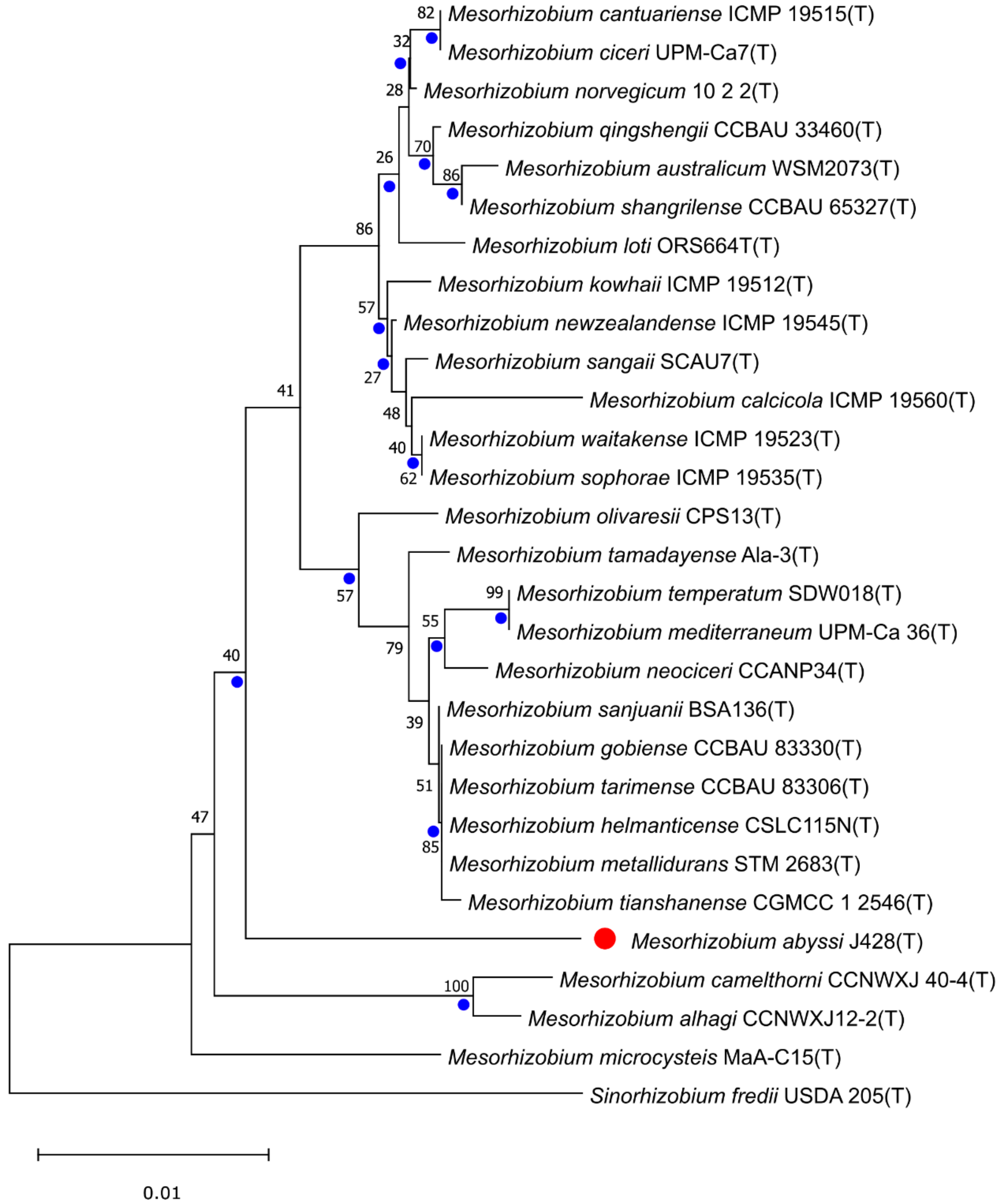

Figure S9: Phylogeny reconstruction of strain J428^{T} and its closest type strains based on secondary structure of 16S rRNA gene. The evolutionary history was inferred using the Neighbor-Joining method (38) based on the Kimura 2-parameter model (39). Bootstrapping was used to test the topology of the tree (1000 replicates), the percentage of trees in which the associated taxa clustered together is indicated. A discrete Gamma distribution was used to model evolutionary rate differences among sites (5 categories). A total of 1,290 positions were used in the final dataset. The blue dots show where evolutionary branches converge, as determined using the Maximum Likelihood method (40) with 1,000 replicates. Evolutionary analyses were conducted in MEGA11 (41). The bar represents the number of substitutions per site.

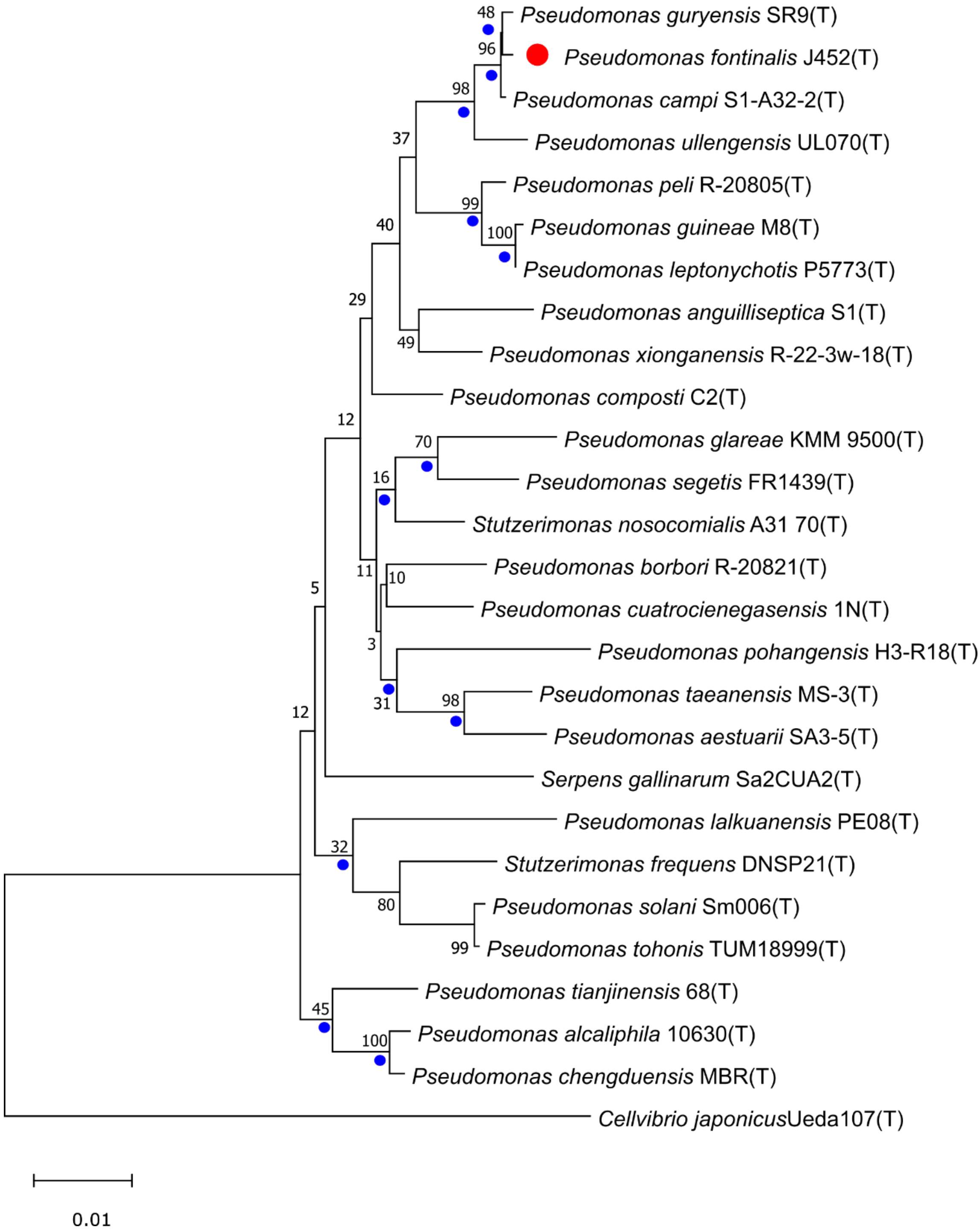

Figure S10: Phylogeny reconstruction of strain J452^{T} and its closest type strains based on secondary structure of 16S rRNA gene. The evolutionary history was inferred using the Neighbor-Joining method (38) based on the Kimura 2-parameter model (39). Bootstrapping was used to test the topology of the tree (1000 replicates), the percentage of trees in which the associated taxa clustered together is indicated. A discrete Gamma distribution was used to model evolutionary rate differences among sites (5 categories). A total of 1,410 positions were used in the final dataset. The blue dots show where evolutionary branches converge, as determined using the Maximum Likelihood method (40) with 1,000 replicates. Evolutionary analyses were conducted in MEGA11 (41). The bar represents the number of substitutions per site.

References

1. Damdintogtokh T, Cha IT, Kim MK. *Hymenobacter guriensis* sp. nov., and *Hymenobacter duratus* sp. nov., radiation-resistant species isolated from soil in South Korea. Curr Microbiol. 2021;78(8):3334-41.
2. Cho ES, Park SL, Nam YD, Lim SI, Kim DY, Jung DH, et al. *Hymenobacter lutimineralis* sp. nov., belonging to the family *Hymenobacteraceae*, isolated from zeolite. Antonie van Leeuwenhoek. 2020;113(7):947-57.
3. Zhang G, Niu F, Busse H-J, Ma X, Liu W, Dong M, et al. *Hymenobacter psychrotolerans* sp. nov., isolated from the Qinghai-Tibet Plateau permafrost region. Int J Syst Evol Microbiol. 2008;58(5):1215-20.
4. Liu K, Liu Y, Wang N, Gu Z, Shen L, Xu B, et al. *Hymenobacter glacieicola* sp. nov., isolated from glacier ice. Int J Syst Evol Microbiol. 2016;66(10):3793-8.
5. Hirsch P, Ludwig W, Hethke C, Sittig M, Hoffmann B, Gallikowski CA. *Hymenobacter roseosalivarius* gen. nov., sp. nov. from continental Antartica soils and sandstone: bacteria of the *Cytophaga/Flavobacterium/Bacteroides* line of phylogenetic descent. Syst Appl Microbiol. 1998;21(3):374-83.
6. Jin L, Ko S-R, Ahn C-Y, Lee H-G, Oh H-M. *Rhizobacter profundi* sp. nov., isolated from freshwater sediment. Int J Syst Evol Microbiol. 2016;66(5):1926-31.
7. Imai S, Yoshida R, Endo Y, Fukunaga Y, Yamazoe A, Kasai D, et al. *Rhizobacter gummiphilus* sp. nov., a rubber-degrading bacterium isolated from the soil of a botanical garden in Japan. J Gen Appl Microbiol. 2013;59(3):199-205.
8. Wei L, Si M, Long M, Zhu L, Li C, Shen X, et al. *Rhizobacter bergeniae* sp. nov., isolated from the root of *Bergenia scopulosa*. Int J Syst Evol Microbiol. 2015;65(2):479-84.
9. Goto M, Kuwata H. *Rhizobacter daucus* gen. nov., sp. nov., the causal agent of carrot bacterial gall. Int J Syst Evol Microbiol. 1988;38(3):233-9.
10. Chen D-Z, Yu N-N, Chu Q-Y, Chen J, Ye J-X, Cheng Z-W, et al. *Piscinibacter caeni* sp. nov., isolated from activated sludge. Int J Syst Evol Microbiol. 2018;68(8):2627-32.
11. Dahal RH, Han JY, Lee H, Chaudhary DK, Kim DU. *Aquabacterium terrae* sp. nov., isolated from soil. Arch Microbiol. 2021;203(6):3183-9.
12. Hirose S, Tank M, Hara E, Tamaki H, Mori K, Takaichi S, et al. *Aquabacterium pictum* sp. nov., the first aerobic bacteriochlorophyll a-containing fresh water bacterium in the genus *Aquabacterium* of the class *Betaproteobacteria*. Int J Syst Evol Microbiol. 2020;70(1):596-603.
13. Khan IU, Habib N, Asem MD, Salam N, Xiao M, Zhou E-M, et al. *Aquabacterium tepidiphilum* sp. nov., a moderately thermophilic bacterium isolated from a hot spring. Int J Syst Evol Microbiol. 2018;69(2):337-42.
14. Stackebrandt E, Verbarg S, Frühling A, Busse H-J, Tindall BJ. Dissection of the genus *Methylibium*: reclassification of *Methylibium fulvum* as *Rhizobacter fulvus* comb. nov., *Methylibium aquaticum* as *Piscinibacter aquaticus* gen. nov., comb. nov. and *Methylibium subsaxonicum* as *Rivibacter subsaxonicus* gen. nov., comb. nov. and emended descriptions of the genera *Rhizobacter* and *Methylibium*. Int J Syst Evol Microbiol. 2009;59(10):2552-60.

15. Elbanna K, Lütke-Eversloh T, Van Trappen S, Mergaert J, Swings J, Steinbüchel A. *Schlegelella thermodepolymerans* gen. nov., sp. nov., a novel thermophilic bacterium that degrades poly(3-hydroxybutyrate-co-3-mercaptopropionate). Int J Syst Evol Microbiol. 2003;53(4):1165-8.
16. Sheu S-Y, Hsieh T-Y, Chen W-M. *Aquincola rivuli* sp. nov., isolated from a freshwater stream. Int J Syst Evol Microbiol. 2019;69(8):2226-32.
17. Lechner U, Brodkorb D, Geyer R, Hause G, Härtig C, Auling G, et al. *Aquincola tertiaricarbonis* gen. nov., sp. nov., a tertiary butyl moiety-degrading bacterium. Int J Syst Evol Microbiol. 2007;57(6):1295-303.
18. Chen WM, Chen YL, Li YS, Sheu SY. *Aquincola amnicola* sp. nov., isolated from a freshwater river. Arch Microbiol. 2018;200(5):811-7.
19. Huq MA, Rahman MS, Rahman MM. *Aquincola agrisoli* sp. nov., isolated from rhizospheric soil of eggplant and in silico genome mining for the prediction of biosynthetic gene clusters. Int J Syst Evol Microbiol. 2024;74(4).
20. Ding Y-P, Khan IU, Li M-M, Xian W-D, Liu L, Zhou E-M, et al. *Calidifontimicrobium sediminis* gen. nov., sp. nov., a new member of the family *Comamonadaceae*. Int J Syst Evol Microbiol. 2019;69(2):434-40.
21. Lingens F, Blecher R, Blecher H, Blobel F, Eberspächer J, Fröhner C, et al. *Phenylobacterium immobile* gen. nov., sp. nov., a gram-negative bacterium that degrades the herbicide chloridazon. Int J Syst Evol Microbiol. 1985;35(1):26-39.
22. Oh YS, Roh DH. *Phenylobacterium muchangponense* sp. nov., isolated from beach soil, and emended description of the genus *Phenylobacterium*. Int J Syst Evol Microbiol. 2012;62(Pt 4):977-83.
23. Farh ME-A, Kim Y-J, Singh P, Hoang V-A, Yang D-C. *Phenylobacterium panacis* sp. nov., isolated from the rhizosphere of rusty mountain ginseng. Int J Syst Evol Microbiol. 2016;66(7):2691-6.
24. Chu C, Yuan C, Liu X, Yao L, Zhu J, He J, et al. *Phenylobacterium kunshanense* sp. nov., isolated from the sludge of a pesticide manufacturing factory. Int J Syst Evol Microbiol. 2015;65(Pt_2):325-30.
25. Baek C, Shin SK, Yi H. *Flavobacterium magnum* sp. nov., *Flavobacterium pallidum* sp. nov., *Flavobacterium crocinum* sp. nov. and *Flavobacterium album* sp. nov. Int J Syst Evol Microbiol. 2018;68(12):3837-43.
26. Dong K, Xu B, Zhu F, Wang G. *Flavobacterium hauense* sp. nov., isolated from soil and emended descriptions of *Flavobacterium subsaxonicum*, *Flavobacterium beibuense* and *Flavobacterium rivuli*. Int J Syst Evol Microbiol. 2013;63(Pt_9):3237-42.
27. Kuo I, Saw J, Kapan DD, Christensen S, Kaneshiro KY, Donachie SP. *Flavobacterium akiainvivens* sp. nov., from decaying wood of *Wikstroemia oahuensis*, Hawai‘i, and emended description of the genus *Flavobacterium*. Int J Syst Evol Microbiol. 2013;63(Pt_9):3280-6.
28. Ali Z, Cousin S, Frühling A, Brambilla E, Schumann P, Yang Y, et al. *Flavobacterium rivuli* sp. nov., *Flavobacterium subsaxonicum* sp. nov., *Flavobacterium swingsii* sp. nov. and *Flavobacterium reichenbachii* sp. nov., isolated from a hard water rivulet. Int J Syst Evol Microbiol. 2009;59(10):2610-7.
29. Cai H, Zeng Y, Wang Y, Cui H, Jiang H. *Flavobacterium cyanobacteriorum* sp. nov., isolated from cyanobacterial aggregates in a eutrophic lake. Int J Syst Evol Microbiol. 2018;68(4):1279-84.

30. Zheng WT, Li Y, Wang R, Sui XH, Zhang XX, Zhang JJ, et al. *Mesorhizobium qingshengii* sp. nov., isolated from effective nodules of *Astragalus sinicus*. Int J Syst Evol Microbiol. 2013;63(Pt 6):2002-7.
31. Lu YL, Chen WF, Wang ET, Han LL, Zhang XX, Chen WX, et al. *Mesorhizobium shangrilense* sp. nov., isolated from root nodules of *Caragana* species. Int J Syst Evol Microbiol. 2009;59(12):3012-8.
32. De Meyer SE, Wee Tan H, Heenan PB, Andrews M, Willems A. *Mesorhizobium waimense* sp. nov. isolated from *Sophora longicarinata* root nodules and *Mesorhizobium cantuariense* sp. nov. isolated from *Sophora microphylla* root nodules. Int J Syst Evol Microbiol. 2015;65(Pt_10):3419-26.
33. De Meyer SE, Tan HW, Andrews M, Heenan PB, Willems A. *Mesorhizobium calcicola* sp. nov., *Mesorhizobium waitakense* sp. nov., *Mesorhizobium sophorae* sp. nov., *Mesorhizobium newzealandense* sp. nov. and *Mesorhizobium kowhaii* sp. nov. isolated from *Sophora* root nodules. Int J Syst Evol Microbiol. 2016;66(2):786-95.
34. Timsy, Spanner T, Ulrich A, Kublik S, Foesel BU, Kolb S, et al. *Pseudomonas campi* sp. nov., a nitrate-reducing bacterium isolated from grassland soil. Int J Syst Evol Microbiol. 2021;71(5).
35. Kim CM, Jeong JW, Lee DH, Kim SB. *Pseudomonas guryensis* sp. nov. and *Pseudomonas ullengensis* sp. nov., isolated from soil. Int J Syst Evol Microbiol. 2021;71(11).
36. Vanparys B, Heylen K, Lebbe L, De Vos P. *Pseudomonas peli* sp. nov. and *Pseudomonas borbori* sp. nov., isolated from a nitrifying inoculum. Int J Syst Evol Microbiol. 2006;56(8):1875-81.
37. Nováková D, Švec P, Zeman M, Busse H-J, Mašlaňová I, Pantůček R, et al. *Pseudomonas leptonychotis* sp. nov., isolated from Weddell seals in Antarctica. Int J Syst Evol Microbiol. 2020;70(1):302-8.
38. Saitou N, Nei M. The neighbor-joining method: a new method for reconstructing phylogenetic trees. Mol Biol Evol. 1987;4(4):406-25.
39. Kimura M. A simple method for estimating evolutionary rates of base substitutions through comparative studies of nucleotide sequences. Journal of molecular evolution. 1980;16(2):111-20.
40. Nei M, Kumar S. Molecular evolution and phylogenetics: Oxford University Press, USA; 2000.
41. Tamura K, Stecher G, Kumar S. MEGA11: Molecular evolutionary genetics analysis version 11. Mol Biol Evol. 2021;38(7):3022-7.
